# Synthesis of Na_3_WH_9_ and Na_3_ReH_8_ Ternary Hydrides at High Pressures

**DOI:** 10.1021/acs.inorgchem.4c02691

**Published:** 2024-10-31

**Authors:** Tomas Marqueño, Israel Osmond, Mikhail A. Kuzovnikov, Hannah A. Shuttleworth, Samuel Gallego-Parra, Eugene Gregoryanz, Andreas Hermann, Ross T. Howie, Miriam Peña-Alvarez

**Affiliations:** † Center for Science at Extreme Conditions (CSEC) and the School of Physics and Astronomy, The University of Edinburgh, Peter Guthrie Tait Road, Edinburgh EH3 9FD, U.K.; ‡ European Synchrotron Radiation Facility (ESRF), Grenoble 38000, France; § Key Laboratory of Materials Physics, Institute of Solid State Physics, HFIPS, Chinese Academy of Sciences, Hefei 230031, China; ∥ Center for High Pressure Science and Technology Advanced Research (HPSTAR), Shanghai 201203, China

## Abstract

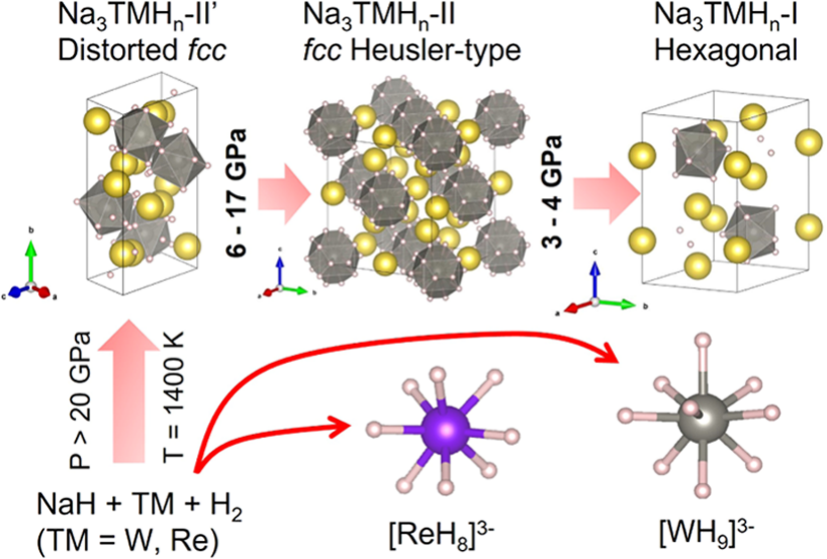

The Na–W–H and Na–Re–H ternary systems were studied in a diamond anvil cell through X-ray diffraction and Raman spectroscopy, supported by density functional theory and molecular dynamics calculations. Na_3_WH_9_ can be synthesized above 7.8 GPa and 1400 K, remaining stable between at least 0.1 and 42.1 GPa. The rhenium analogue Na_3_ReH_8_ can form at 10.1 GPa upon laser heating, being stable between at least 0.3 and 32.5 GPa. Na_3_WH_9_ and Na_3_ReH_8_ host [WH_9_]^3–^ and [ReH_8_]^3–^ anions, respectively, forming homoleptic 18-electron complexes in both cases. Both ternary hydrides show similar structural types and pressure dependent phase transitions. At the highest pressures they adopt a distorted fcc Heusler structure (Na_3_WH_9_–II′ and Na_3_ReH_8_–II′) while upon decompression the structure symmetrizes becoming fcc between ∼6.4 and 10 GPa for Na_3_WH_9_–II and at 17 GPa for Na_3_ReH_8_–II. On further pressure release, the fcc phases transform into variants of a (quasi-) hexagonal structure at ∼3 GPa, Na_3_WH_9_–I and Na_3_ReH_8_–I.

## Abstract 2

In this work we explore the synthesis of Na_3_WH_9_ and Na_3_ReH_8_ at pressures over 20 GPa using diamond anvil cells and laser heating techniques. These compounds host 18-electron [WH_9_]^3−^ and [ReH_8_]^3−^ homoleptic anions, respectively. The samples were analyzed using X-ray diffraction and Raman spectroscopy. At the highest studied pressures, these compounds crystallize in a distorted fcc structure. Upon decompression, the distortion disappears and below 3–4 GPa they transform into variants of a hexagonal phase.

## Introduction

The search for novel high-temperature superconductors has motivated the study of hydrogen-rich systems under high pressures, leading to the discovery of new binary metal–hydrogen compounds.^1^ Some of these novel materials, such as LaH_10_,^2^ CaH_6_^3,4^ or H_3_S^5,6^ exhibit superconducting properties with high critical temperature (*T*_c_) above 150 GPa. Despite their promising attributes, such binary hydrides tend to decompose upon decompression, complicating the attainment of superconductive phases under ambient conditions.^7−9^

The study of ternary hydrides at high pressures has been suggested as an alternative approach toward achieving superconductivity at lower pressures.^10−12^ This is because, the addition of a third element is expected to stabilize superconducting phases at lower pressures.^10,12−15^ In fact, theoretical studies have predicted superconducting properties in compounds combining alkaline/alkaline earth metals with transition metal (TM) and hydrogen (complex transition metal hydrides, (CTMHs)), such as K_2_ReH_9_ (*T*_c_ = 127.1 K at 75 GPa)^16^ or Mg_2_IrH_6_ (*T*_c_ = 160 K at ambient pressure).^10^ However, experimental research has shown modest *T*_c_ values for CTMHs such as BaReH_9_^17^ and Li_5_MoH_11_^18^ (above 100 GPa and below 10 K).

In CTMHs the TM atoms are coordinated by an unusually high number of hydrogen atoms forming homoleptic complex ions, such as [OsH_7_]^3–^,^19^ [NbH_9_]^4–^,^20^ and [NiH_5_]^3–^.^21^ Traditionally, the synthesis of CTMHs has been conducted using autoclave techniques, where a mixture of active (alkali, alkaline earth or rare earth) and transition metals react at room pressure in a hydrogen atmosphere at high temperatures (300–700 °C).^13,22^

Historically, TMs from groups 3 to 6 were thought incapable of forming semiconducting CTMHs.^23^ However, it was later shown that the use of large volume presses to 5 GPa and from 300 to 800 °C with AlH_3_ as hydrogen precursor, leads to the synthesis of compounds such as Mg_3_CrH_8_,^24^ Li_5_(TM)H_11_ (TM = Mo, W) and Li_6_(TM)H_11_ (TM = Nb, Ta).^20^ All these compounds contain interstitial H^–^ anions as well as TMH_*n*_ homoleptic ionic complexes.

TM–H hydrides containing Na cations support high-coordinated TM complexes without interstitial H^–^.^14,21^ For TMs in groups 8, 9, and 10 these hydrides display a rich polymorphism in phases recovered to ambient conditions, with a *Pnma* structure type for Na_3_NiH_5_^21^ and Na_3_(TM)H_6_ (TM = Co, Rh, Ir)^25^ and a *P*4_2_/*mnm* structure type for Na_3_(TM)H_7_ (TM = Fe, Ru, Os).^14^ In these CTMHs the hydrogen content decreases with the TM group number; therefore, Na–TM–H with TMs from groups 6 and 7 could yield higher hydrogen-to-metal ratios (H to M ratio), which is beneficial for potential hydrogen storage applications. Under pressure, the Na-based CTMHs take up fcc-like structures with the metal atoms on Heusler sites, and rotationally disordered TMH_*n*_ complexes.

In this study, the synthesis of two novel Na–Re–H and Na–W–H compounds under high temperatures and pressures, using diamond anvil cells and infrared laser heating, are reported. Na_3_WH_9_ and Na_3_ReH_8_ form from NaH, W/Re and H_2_ upon laser heating above 7.8 and 10.1 GPa, respectively, remaining stable between at least 30 GPa and down to <0.5 GPa. X-ray diffraction (XRD) and Raman spectroscopy, supported by density functional theory (DFT) calculations are used for the characterization of the new compounds. XRD measurements show that Na_3_WH_9_–II′ adopts a distorted fcc Heusler structure^26^ above 20 GPa (the first identified rotationally ordered phase), containing [WH_9_]^3–^ units. Upon decompression, below 10 GPa, Na_3_WH_9_–II′ symmetrizes into an fcc structure (Na_3_WH_9_–II), which then transitions into an hexagonal structure, Na_3_WH_9_–I near ∼3 GPa.^26^ Na_3_ReH_8_, which also adopts a Heusler structure with a small distortion, was synthesized from NaH + Re + H_2_ at 32.5 and 25.5 GPa using laser heating to 1400 K. Na_3_ReH_8_ features stable 18-electron [ReH_8_]^3–^ complexes. Upon decompression, XRD measurements show that Na_3_ReH_8_ also undergoes a series of analogous phase transitions at 17 and 3.5 GPa. The Raman spectra of these phases present broad bands near 1100 and 2000 cm^–1^, associated with TM–H bending and stretching modes of the [TMH_*n*_]^−3^ units, respectively. DFT ab initio calculations were used to evaluate the relative stability of the different structural phases for both ternary hydrides, reproducing the experimentally observed phase transitions. Additionally, molecular dynamics (MD) calculations captured the pseudorotational character of the Na_3_WH_9_–II and Na_3_ReH_8_–II structures, with hydrogen atoms occupying nonisotropic shells around the TM.

## Results

### Powder X-ray Diffraction Experiments

**Figure 1:**
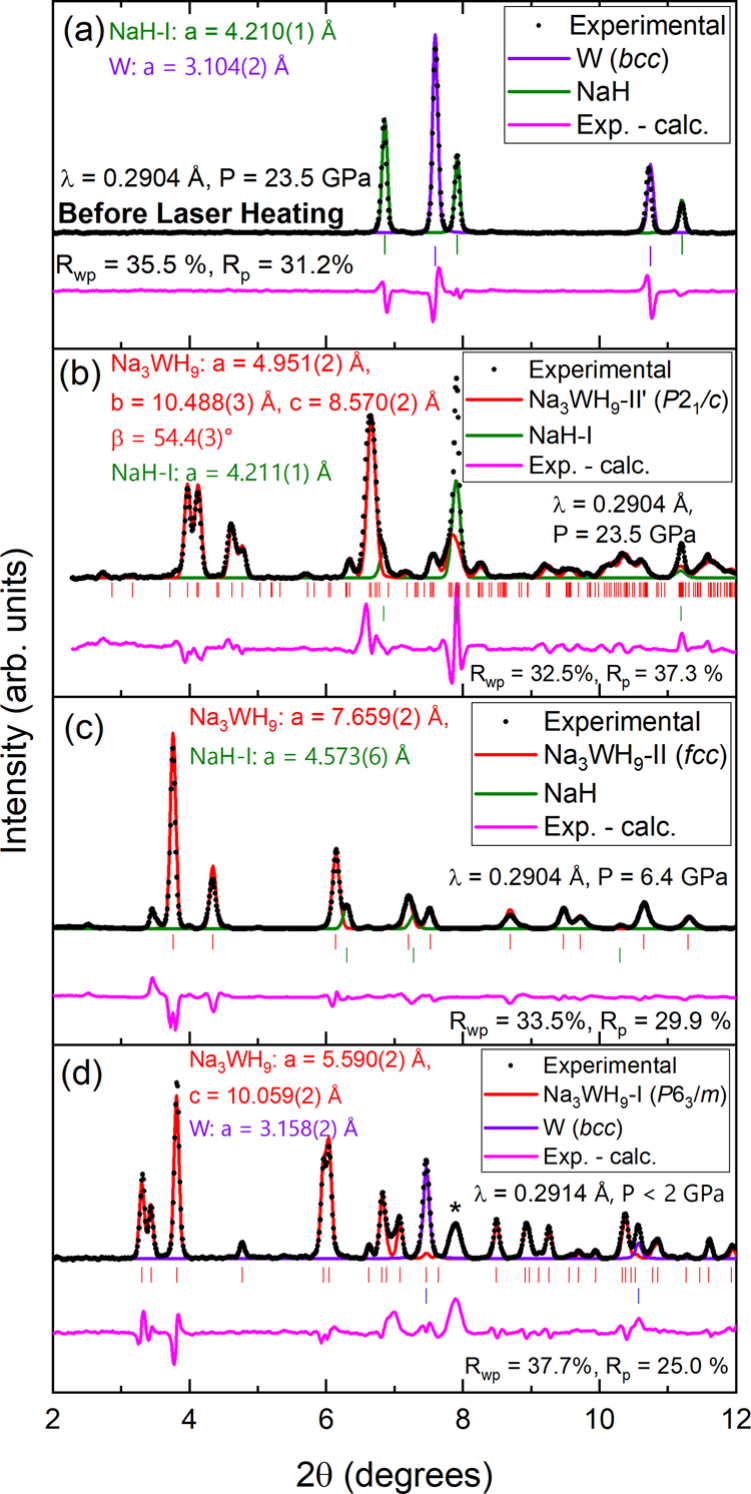
Rietveld refinements of powder XRD patterns at different pressures in two different experiments (λ = 0.2904 and 0.2914 Å). (a, b) Show the sample before and after laser heating, respectively. (c, d) Correspond to different phases observed upon decompression. The reflection labeled with an asterisk is associated with the rhenium gasket. Experimental data is depicted with black dots. The difference between the experimental and calculated pattern is depicted with a pink line. The calculated pattern for each individual phase is represented with its corresponding colored line, which is indicated on the legend. the March–Dollase model was used to account for preferred orientations. Pressures and both *R*_wp_ and *R*_p_ factors are shown on each plot. The calculated positions for the Bragg reflections of each phase are depicted with vertical ticks. The refined parameters for the Na_3_WH_9_ phases shown in (b–d) are shown in Tables S1–S3, respectively.

Approximately two parts sodium hydride (NaH) to one part tungsten (W) by volume were loaded in a diamond anvil cell (DAC) and clamped with excess hydrogen (H_2_) at 0.2 GPa. As shown in Figures 1(a,b) and S1–S3 the XRD patterns of the mixture fully transform during laser heating at 23.5 and 20 GPa (to 1400 K^27^). The new diffraction patterns are compatible with a distorted fcc Heusler arrangement,^26^ with the Na and W atoms forming a metallic sublattice that can be described by either the space group *Immm* or *Pmnm* (Figure S2(a,b), respectively) in a proportion of 3:1 (Na_3_WH_*n*_). These are the highest symmetry subgroups of the fcc structure that are consistent with the experimental data presented here. Nevertheless, the stability of these structures is compromised when hydrogen atoms are incorporated into the DFT calculations. We performed DFT-based search for a possible arrangement of hydrogen atoms in this material, and found a possible candidate with the composition of Na_3_WH_9_ with a monoclinic crystal structure and space group *P*2_1_/*c*. Rietveld refinements (Figure 1 (b)) of the crystal structure of the sample after laser heating at 23.5 GPa corroborate that this Na_3_WH_9_–II′ phase can be indexed to the monoclinic (*P*2_1_/*c*) structure (*a* = 4.951 Å, *b* = 10.488(3) Å, *c* = 8.570(2) Å and β = 54.4(3) °C; *R*_wp_ = 32.5%). We will refer to this distorted cubic phase as Na_3_WH_9_–II′. Similarly, when the NaH–W–H_2_ sample is compressed to 33.5 GPa and laser heated to 1400 K, Na_3_WH_9_–II′ is also formed (Figure S4(a,b)). Upon compression up to 42 GPa, Na_3_WH_9_–II′ remains stable and no features of other Na–W–H phases are observed (Figure S5(c)).

Upon decompression of Na_3_WH_9_–II′, we observe the merging of some of its Bragg reflections at 6.4 GPa (Figures 1(c) and S1 and S5), indicating that the structure undergoes a phase transition to fcc Na_3_WH_9_–II, a more symmetric structure. At 5.5 GPa the XRD patterns are dominated by Na_3_WH_9_–II. When Na_3_WH_9_–II is further decompressed to 3 GPa, it undergoes a phase transition to Na_3_WH_9_–I, adopting a hexagonal structure (Figure 1(d)).^26^ The patterns of Na_3_WH_9_–I can be indexed to a 3Na:W metallic sublattice with space groups *P*6_3_/*mmc*^26^ or *P*6_3_/*cm*,^28^ similar to those reported for Na_3_As at low pressures. After considering hydrogen occupancies, DFT calculations reduce the total symmetry of the crystal to *P*6_3_/*m*. A Rietveld refinement using a structure with space group *P*6_3_/*m* shows good compatibility with the experimental data (*a* = 5.590(2), *c* = 10.059(2) Å; *R*_wp_ = 37.7%), Figure 1 (d). All the phases found for Na_3_WH_9_–II′ (42–6.4 GPa), -II (6.4–3 GPa) and -I (below 3 GPa) are characterized by [WH_9_]^3–^ anionic units. These accommodate 18 valence electrons, with tungsten achieving its highest (and most common) oxidation state (+6).

**Figure 2:**
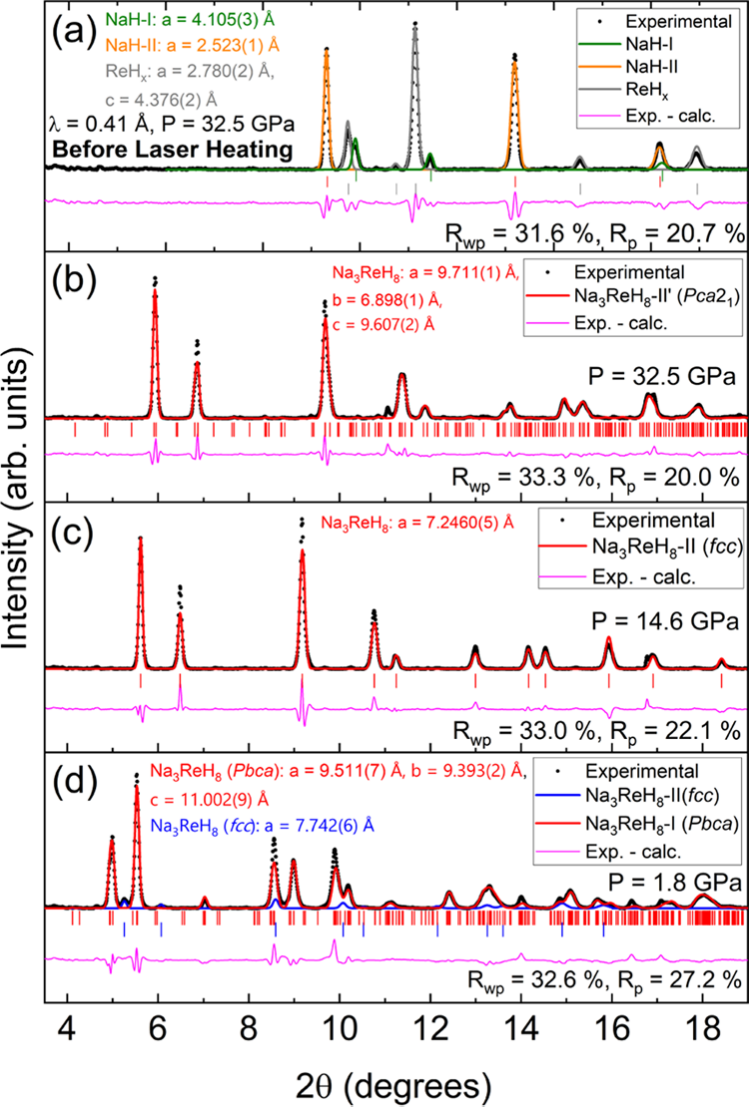
XRD patterns and corresponding Rietveld refinements of the Na–Re–H sample before laser heating (a), after laser heating (b) and subsequently decompressed down to 14.6 (c) and 1.8 GPa (d). Experimental data is represented with black dots. The difference between these and the refinement is depicted with a pink solid line. The rest of the colored solid lines correspond to the calculated partial contributions the phases indicated on the legends. Vertical ticks denote the position of the Bragg reflections. The refined parameters for the Na_3_ReH_8_ phases shown in (b–d) are presented in Tables S4–S6, respectively.

In a different set of experiments, we loaded NaH, Re and H_2_ gas at 0.2 GPa in a DAC. The sample was compressed to 32.5 GPa and laser-heated using the rhenium metal as a coupler. XRD patterns of the sample before and after heating are shown in Figure 2(a,b). As in Na_3_WH_9_–II′, the diffraction peak distribution of the product, Na_3_ReH_8_–II′, resembles those of an fcc lattice, the Bragg peaks of the phase synthesized after laser heating at 32.5 GPa are not symmetric and can be more accurately described by an orthorhombic distortion of the structure. In fact, our DFT calculations reduce the fcc Heusler of Na_3_ReH_8_ to a slightly distorted variant, which shows a reasonably good compatibility with our data, as shown in the Rietveld refinement depicted in Figure 2 (b) (*Pca*2_1_; *a* = 9.711(1) Å, *b* = 6.898(1) Å and *c* = 9.607(2) Å; *R*_wp_ = 33.3%). We suggest the formula Na_3_ReH_8_, as only in the [ReH_8_]^3–^ ions can accommodate 18 valence electrons. Hence, whereas W is in its highest oxidation state +6, Re can only achieve its +5 oxidation state in Na–TM–H compounds.

As in Na_3_WH_9_, Na_3_ReH_8_–II′ transitions to fcc Na_3_ReH_8_–II (Figures 2(c) and S6(a,b): upon decompression at around 17 GPa, the (020) and (202) reflections of Na_3_ReH_8_–II′ merge into a single (200) reflection characteristic of an fcc phase). At 1.8 GPa, the XRD patterns of Na_3_ReH_8_ present a different peak distribution that corresponds to a new low-pressure phase Na_3_ReH_8_–I, Figure 2(d). Similarly to Na_3_WH_9_–I, we initially considered a highly symmetric hexagonal lattice for Na_3_ReH_8_–I, which our DFT calculations reduced to a *Pbca* symmetry. Rietveld refinements in Figure 2(d) show a reasonably good compatibility with this structural model (*a* = 9.511(7) Å, *b* = 9.393(2) Å, *c* = 11.002(9) Å; *R*_wp_ = 32.6%). In this structure, the Re atoms lay on a quasi-hexagonal sublattice, similar to that of Na_3_WH_9_–I. Laser heating the reactants at 25.5 GPa yields identical products and phase transformations under decompression (see Figure S6(c)).

**Figure 3:**
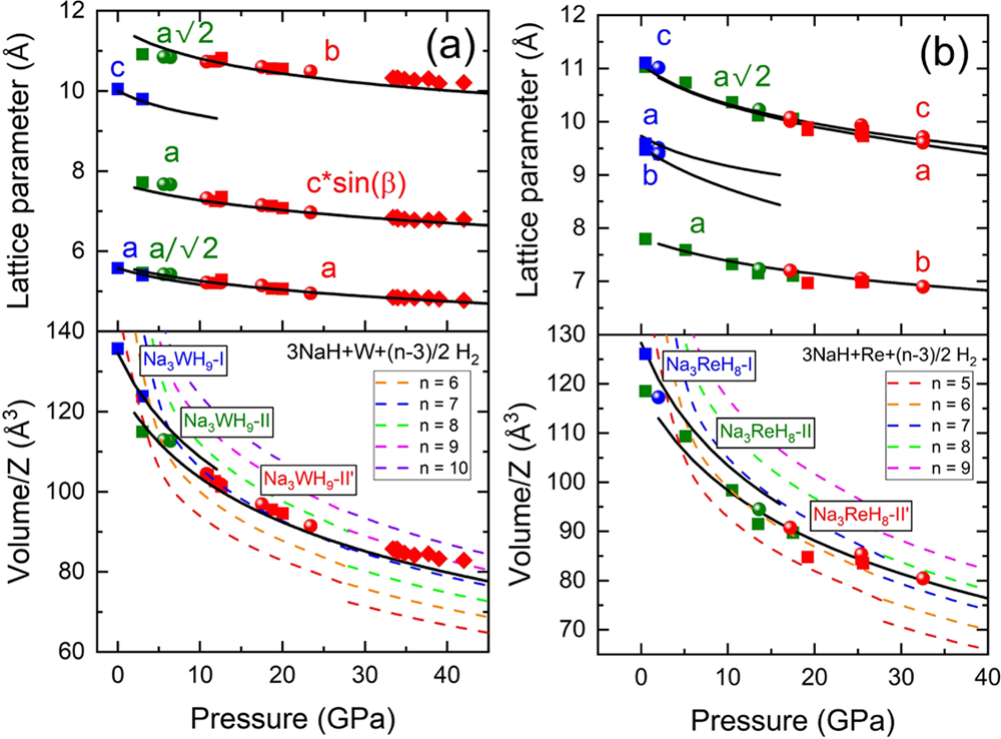
Pressure dependence of the lattice parameters and volumes per formula unit of (a) Na_3_WH_9_ and (b) Na_3_ReH_8_. Circles, squares and diamonds correspond to the data collected during different sets of experiments. The II′, II, and I phases are indicated with red, green and blue symbols, respectively. Our calculated DFT values are depicted with black solid lines. Colored dashed lines correspond to the sum of the reactant volumes (NaH, TM, H_2_)^29−32^ for different quantities of hydrogen (values of *n*). The discontinuity in these lines correspond to the NaH-I to NaH-II phase transition at ∼29 GPa.^29^

Figure 3(a,b) present lattice parameters and unit-cell volumes per formula unit as a function of pressure for the Na_3_WH_9_ and Na_3_ReH_8_ phases. The unit-cell volumes are compared with the sum of the individual volumes of the reactants, considering different hydrogen amounts.^29−32^ In all cases, the experimental volumes for both Na–W–H and Na–Re–H compounds are smaller than the sums of the individual volumes of the reagents i.e., all the suggested stoichiometries fulfill Le Châtelier’s principle regarding volume change upon reaction. Despite hydrogen disorder is expected in the fcc phases, the lattice parameters of the H-ordered phases are in reasonably good agreement with the experimental results (Figure 3). The subgroup axis transformation relating the *P*2_1_/*c* space group to the Heusler structure is (*a*, *b*, *c* sin β) = (*a*_fcc_/√2, *a*_fcc_√2, *a*_fcc_). For *Pca*2_1_, this relationship is (*a*, *b*, *c*) = (*a*_fcc_√2, *a*_fcc_, *a*_fcc_√2). Random preferred orientations in our patterns lead to some deviations between the refined (Tables S1–S11) and DFT calculated (Tables S12–S15) atomic coordinates at similar pressures.

**Figure 4:**
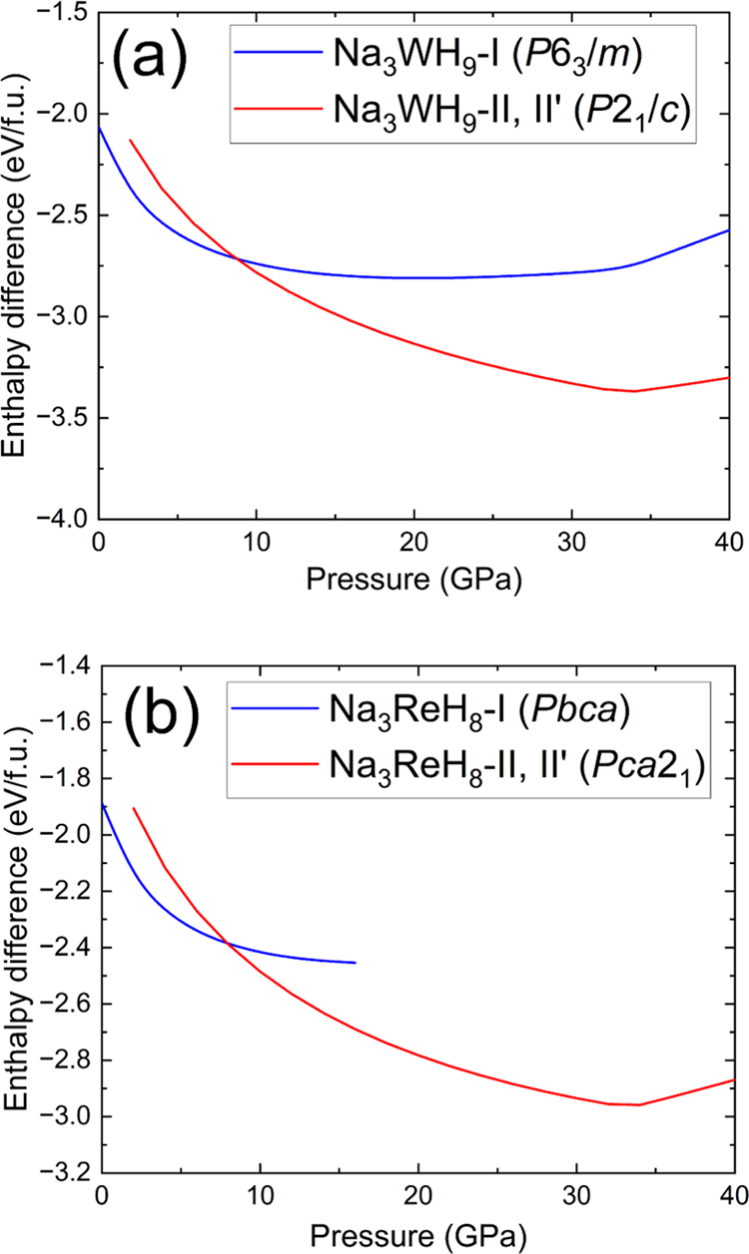
Δ*H* vs *P* curves of the (a) Na–W–H and (b) Na–Re–H systems. The color corresponding to each phase is indicated in the legend. The difference in enthalpy was calculated with respect to the enthalpy of the separate reactants i.e., *H*(NaH) + *H*(TM) + *H*(H_2_). Enthalpy units are given in eV per formula unit (f.u.).

Figure 4 shows Δ*H*(*P*), the relative enthalpy curves between 0 and 40 GPa for the Na–W–H and Na–Re–H systems calculated with DFT. The formation enthalpies of the reaction products were calculated with respect to the sum of the enthalpies of NaH, TM (W, Re) and H_2_. The dynamic stability of the II′ and I phases of Na_3_WH_9_ and Na_3_ReH_8_ was confirmed by DFT (see calculated phonon bands in Figure S7). The fcc structures Na_3_WH_9_–II and Na_3_ReH_8_–II can only be stabilized dynamically, i.e., through MD simulations, therefore DFT calculations at *T* = 0 K reduce the Heusler fcc structure to the *P*2_1_/*c* (*Pca*2_1_) symmetry. For the Na–W–H system, Na_3_WH_9_ is predicted to have lower enthalpy than its separate reactants across the considered pressure range (*P* < 40 GPa). Below 8 GPa, the hexagonal Na_3_WH_9_–I (*P*6_3_/*m*) phase becomes more stable than the Na_3_WH_9_–II′ (distorted cubic *P*2_1_/*c*) phase, in good agreement with the experimental transition between 5.5 and 3 GPa during decompression. In the Na–Re–H system, (Figure 4(b)) both Na_3_ReH_8_–I (*Pbca*) and Na_3_ReH_8_–II′ (*Pca*2_1_) show negative values of Δ*H* in the 0 to 40 GPa range. DFT predicts the transition from the high- to the low-pressure phase below 9 GPa, supporting our experimental observations. Interestingly, Na_3_WH_9_–I and Na_3_ReH_8_–I both exhibit negative values of Δ*H* at low pressures, indicating their stability at ambient conditions. However, relative Gibbs free energies Δ*G* (not calculated here), particularly when considering hydrogen, might favor their decomposition. Unfortunately, the product reacts with the moisture in the air when the DAC is opened, so its stability at ambient conditions was not explored.

**Figure 5:**
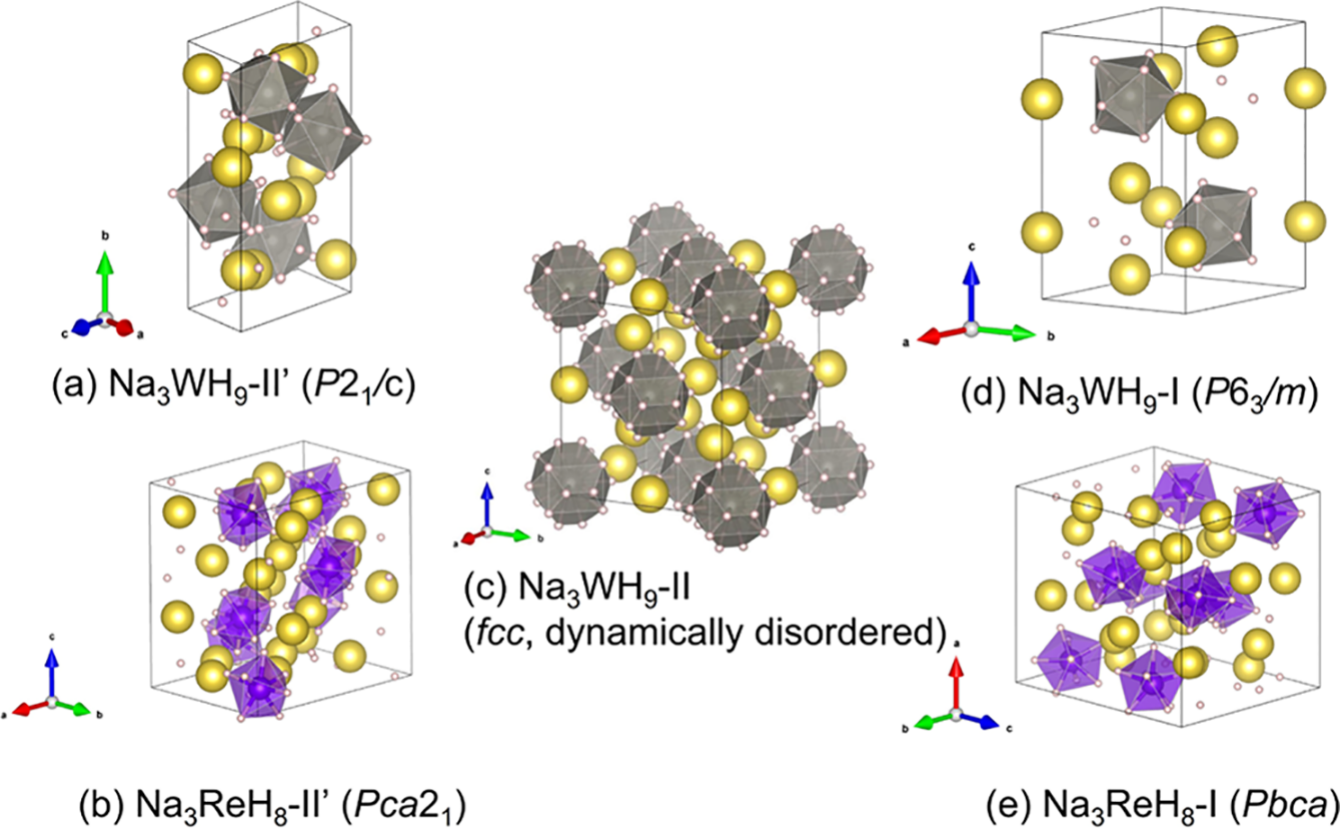
Crystal structures of Na_3_WH_9_ and Na_3_ReH_8_ obtained in calculations: (a) Na_3_WH_9_–II′ (*P*2_1_/*c*, DFT), (b) Na_3_ReH_8_ (*Pca*2_1_, DFT), (c) Na_3_WH_9_–II (fcc, MD), (d) Na_3_WH_9_–I (*P*6_3_/*m*, DFT) and (e) Na_3_ReH_8_–I (*Pbca*, DFT). Yellow, gray, purple and white spheres represent Na, W, Re, and H atoms, respectively. Gray and purple polyhedra represent [WH_9_]^3–^ and [ReH_8_]^3–^.

The DFT calculated crystal structures of the high pressure phases, Na_3_WH_9_–II′ (*P*2_1_/*c*) and Na_3_ReH_8_–II′ (*Pca*2_1_), (assuming static positions for hydrogen atoms i.e., full occupancies) is depicted in Figure 5(a,b), respectively. Na_3_WH_9_–II′ consists of an arrangement of Na^+^ cations and [WH_9_]^3–^ anionic units, in which the Na and W atoms form a slightly distorted Heusler fcc structure. Crystallographic data for the high-pressure phases Na_3_WH_9_–II′ and Na_3_ReH_8_–II′ obtained from DFT calculations are given in Tables S12 and S13, respectively.

Figure 5(c) shows the unit-cell corresponding to the Na_3_WH_9_–II structure (fcc) obtained after MD calculations in which H atoms have 1/2 partial occupations. This Heusler arrangement (fcc) has been reported for Na_3_FeH_7_, Na_3_Co*H*_6_, Na_3_NiH_5_ and K_3_ReH_6_.^14,21,33^ As shown in Figure S8(a), hydrogen atoms are delocalized around the transition metal atoms, occupying spherical shells due to rotation of the [WH_9_]^3–^ anions. The anion complex’ rotations are not fully free, and hydrogen angular distribution is uneven. The calculated time evolution of the lattice parameters for this phase is depicted in Figure S8(b,c), showing how the structure reduces into an fcc lattice with time. Our MD calculations show the equivalent time evolution of the lattice parameters of Na_3_ReH_8_–II at 0, 4, 10, and 20 GPa in Figure S9. These results indicate that pressure reduces hydrogen mobility, hindering rotational degrees of freedom, eventually causing the transition from the rotationally disordered fcc (II) to the distorted high-pressure phases (II′) in both Na_3_WH_9_ and Na_3_ReH_8_.

The Na_3_WH_9_–I phase (*P*6_3_/*m*) shown in Figure 5(d) can be described as an arrangement of two types of linear structures disposed along the *c*-axis direction: pure Na chains and (−Na–Na–[WH_9_]^3–^)– chains. The WH_9_ complexes form a hexagonal close packed structure, with the Na atoms from the pure Na chains occupying centers of the in-planar triangles located in between the two octa-sites; and the remaining Na atoms occupying tetrahedral interstitial sites. The Na_3_ReH_8_–I phase (*Pbca*), whose unit-cell is depicted in Figure 5(e), can be seen as a 4-fold supercell of the hexagonal structure, for instance as (*a*_o_, *b*_o_, *c*_o_) = (*a*_h_ + 2*b*_h_, *c*_h_, 2*a*_h_). DFT calculated crystallographic data for the two phases is given in Tables S14 and S15. The symmetry-lowering in Na_3_ReH_8_–I manifests in the metal lattice as in-plane displacement of the tetrahedral site Na atoms. We performed DFT-MD simulations of Na_3_ReH_8_–I at 2 GPa and noticed that this displacement disappeared upon heating when (simultaneously) the [ReH_8_]^3–^ units became rotationally disordered (Figure S10). Hence, we expect that at low pressure but high temperature, Na_3_ReH_8_ also takes up a hexagonal phase; at low temperature, a disorder–order transition occurs as the [ReH_8_]^3–^ complex is not compatible with hexagonal symmetry.

### Raman Spectroscopy Experiments

**Figure 6:**
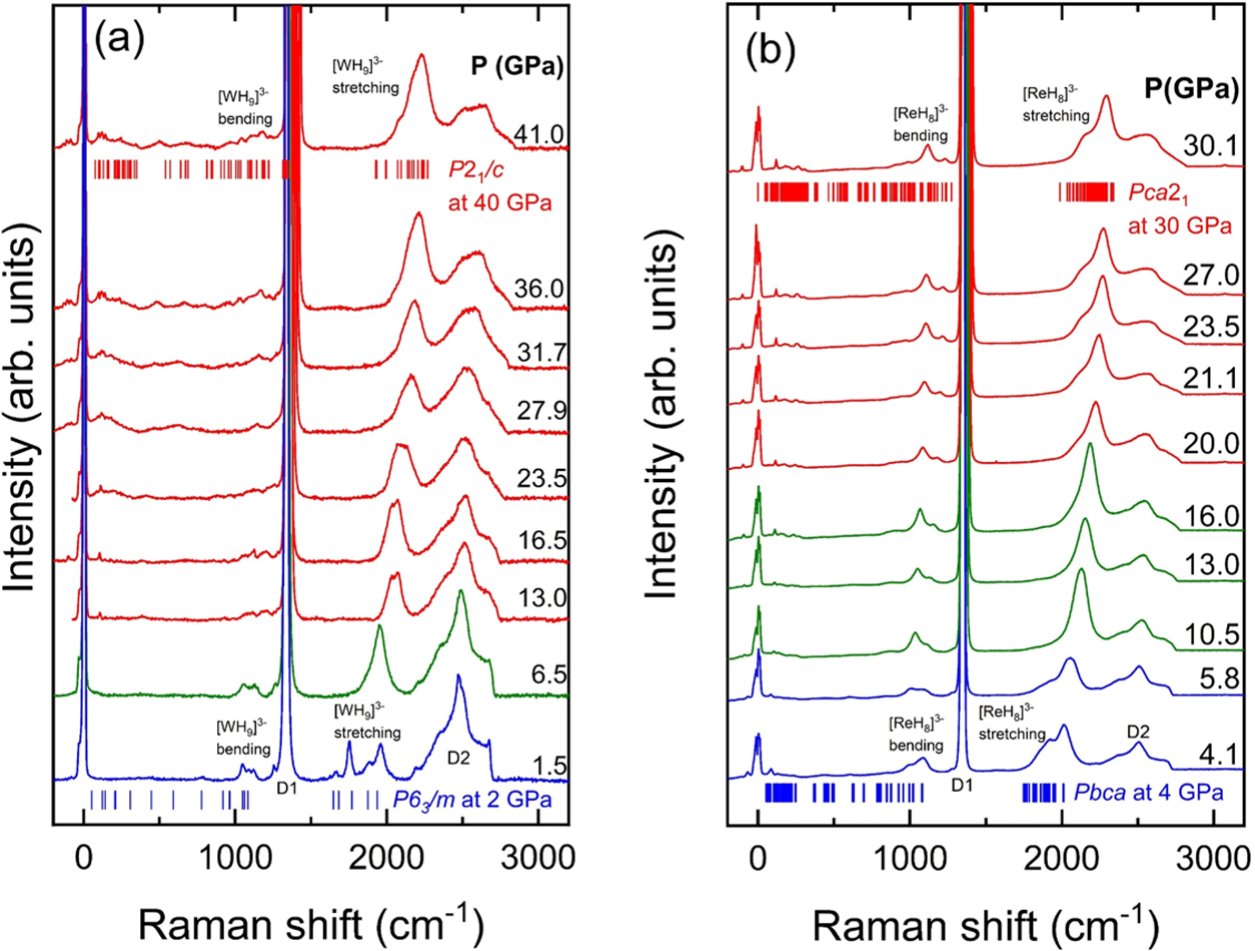
Raman spectra of the (a) Na_3_WH_9_ (decompression) and (b) Na_3_ReH_8_ (compression) phases. The spectra corresponding to the II′, II, and I phases are depicted with red, green and blue lines, respectively. The bands indicated with the labels “D1” and “D2” correspond to the first and second Raman modes of diamond. Vertical ticks stand for the calculated frequencies of the phases indicated in the figure.

As illustrated in Figures 6(a) and S11(a), the Raman spectra of Na_3_WH_9_–II′ exhibit prominent bands in the 1000–1300 and 1900–2500 cm^–1^ ranges, which correspond to the bending and stretching modes of the [TMH_*n*_]^3–^ anionic units.^17,20,34−36^ Particular attention is given to the 1900–2500 cm^–1^ region, indicative of the TM–H stretching mode. Density functional theory (DFT) predicts that the Raman spectra are composed of numerous closely spaced contributions. By analyzing the second derivative of the experimentally obtained spectra at 41 GPa, we identified three distinct components for Na_3_WH_9_–II′ (see Figure S12(a)). Upon decompression from 16.5 to 6.5 GPa, significant spectral changes are observed in the TM–H stretching modes, transitioning from three distinct components to one intense peak with a low-frequency shoulder. This is related with the Na_3_WH_9_–II′ to Na_3_WH_9_–II phase transition. Further decompression to 3.5 GPa results in an additional splitting of these TM–H stretching modes into five components, Figure S12(c), which is attributed to the transition to the Na_3_WH_9_–I phase, which aligns well with the DFT-predicted modes illustrated in Figure 6(a).

Analogous to the Na–W–H system, the Raman spectra of Na_3_ReH_8_–II′ exhibit two principal Raman active regions around 1100 and 2100 cm^–1^, corresponding to the transition metal–hydrogen (TM–H) bending and stretching modes, respectively (Figures S11(b) and 6(b)). Similar to Na_3_WH_9_–II′, the Raman spectra of Na_3_ReH_8_–II′ at approximately 30 GPa in the TM–H stretching region are characterized by multiple contributions predicted by DFT. Through second derivative analysis of the experimental spectrum, we resolved three contributions (Figure S12(d)). Additionally, between 20 and 16 GPa, coinciding with the phase transition to Na_3_ReH_8_–II, the spectra simplify, particularly in the TM–H modes region, which reduce from three to two contributions (Figure S12(e)). During compression from 5.8 to 10.5 GPa (Figure 6(b)) and decompression from 3.7 to 3.2 GPa (Figure S12(b)), coinciding with the phase transition to Na_3_ReH_8_–I, the spectra undergo notable changes. These changes are especially relevant in the TM–H stretching region, which becomes more complex with over ten contributions predicted by DFT, experimentally resolved as three (Figure S12(f)).

**Figure 7:**
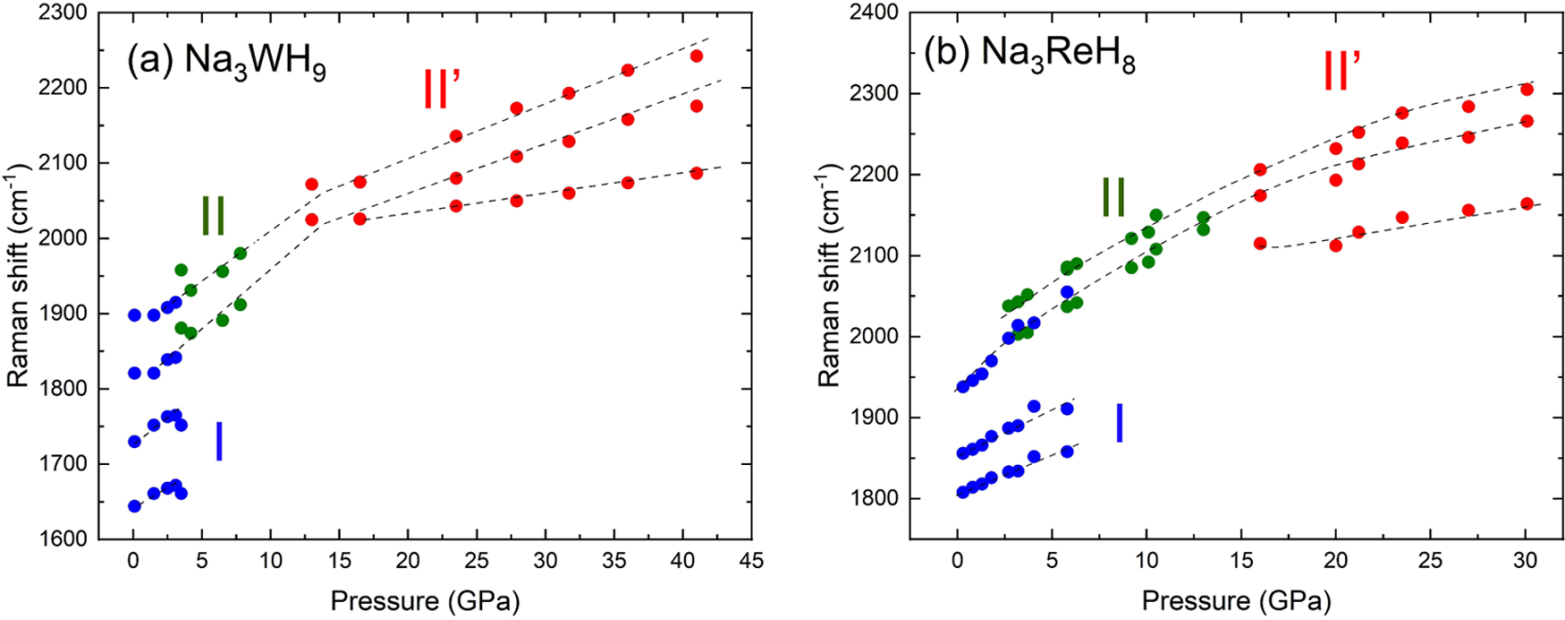
Pressure dependence of the individual Raman bands composing the [TMH_*n*_]^3–^ stretching modes of (a) Na_3_WH_9_ and (b) Na_3_ReH_8_. Dashed lines are a guide to the eye.

The pressure evolution of the TM–H stretching modes contributions is depicted in Figure 7(a),(b) for Na_3_WH_9_ and Na_3_ReH_8_, respectively. There is no significant effect of the transition metal (TM) on the Raman shift of the TM–H stretching modes, as both Re and W are located in the same spectral region. Consistent with other ternary transition metal polyhydrides, regardless of the phase, all spectral contributions in both Na_3_WH_9_ and Na_3_ReH_8_ upshift with pressure, which is related to the shortening of the TM–H bond under pressure.^12,20^ The lower frequency of the TM–H stretching modes associated with the low-pressure hexagonal phases, Na_3_WH_9_–I and Na_3_ReH_8_–I, is related to a decrease in the electronic density of the TM–H bond, leading to an expansion of the [TMH_*n*_]^3–^ unit. A similar analysis was carried out for the H–TM–H bending modes of Na_3_WH_9_ and Na_3_ReH_8_, shown in Figure S13(a,b), respectively.

To explore the stability of Na_3_WH_9_, a NaH–W–H_2_ sample was laser heated at different pressures. Laser heating at 1 GPa and 1400 K did not lead to any spectral changes, indicating no reaction between the precursors. However, laser heating to approximately 1400 K at 7.8 GPa induced spectral changes related to the formation of Na_3_WH_9_–II, as shown in Figure S14(a). Analogous experiments were conducted for the NaH–Re–H_2_ system. Laser heating at 1 GPa and 1400 K did not result in the formation of Na_3_ReH_8_. It was only at pressures above 10 GPa and temperatures of 1400 K that the spectral features of Na_3_ReH_8_–II were identifiable upon quenching, Figure S14(b).

### Discussion

The synthesis of Na_3_WH_9_ and Na_3_ReH_8_ demonstrates the feasibility of forming Na–TM–H compounds with TM of groups 6 and 7 using high pressures and laser heating techniques. Stable novel Na–TM–H compounds featuring *d*^0^ [WH_9_]^3–^ and *d*^2^ [ReH_8_]^3–^ homoleptic anions are proven. These anionic units accommodate 18 valence electrons and may be common to other Na–TM–H compounds with TMs of groups 6 and 7. [ReH_8_]^3–^ represents an interesting case, as this coordination is highly unusual in CTMHs, in which the highest coordination of TM elements is usually either 7 or 9.^13^ In fact, only few systems are known to host 8-coordinated TM atoms, such as Cs_3_OsH_9_ and Rb_3_OsH_9_.^19,37,38^ Other systems with similar stoichiometries to Na_3_ReH_8_, such as Mg_3_CrH_8_ host [CrH_7_]^5–^ anions and interstitial H^–^.^24,25^

As mentioned in Na_3_WH_9_–II and Na_3_ReH_8_–II, the [WH_9_]^3–^ and [ReH_8_]^3–^ anions are in freely rotating states. Low temperatures and high pressures can induce similar distortions, as both restrict dynamic effects. For instance, it has been observed that in Na_3_NiH_5_ the fcc structure observed at 5 GPa and 700 K becomes a quasi-cubic orthorhombic structure when quenched at room temperature. Likewise, reversible transitions from rotational to nonrotational states are the foundation of thermal energy storage in solid–solid phase change materials (ss-PCM’s),^39,40^ which in some cases exhibit crystallographic transformations similar to those seen in the hydride systems in this study.^41^

Both, Na_3_WH_9_ and Na_3_ReH_8_ remain transparent within the whole experimental pressure range. To check for possible metallic phases, we calculated the pressure evolution of the electronic band gap of Na_3_WH_9_–II′ and Na_3_ReH_8_–II′, (Figure S15). The band gap of Na_3_WH_9_–II′ (*P*2_1_/*c*) is predicted to progressively close with increasing pressure, becoming metallic above 180 GPa. Na_3_ReH_8_–II′ is expected to have a smaller band gap than Na_3_WH_9_–II′ below 100 GPa, but it would only decrease substantially above 350 GPa, suggesting that metallicity can only be achieved at extremely high pressures (over 500 GPa). The main reason for the lack of metallicity at more attainable pressures is that [TMH_*x*_]^3–^ anions are disconnected and considerably separated from each other inside the reported structures, which prevents the formation of a monatomic hydrogen framework.^2^

## Conclusions

By combining both XRD and Raman scattering techniques we explore the Na–W(Re)–H ternary systems under pressure. We observed the formation of distinct high-hydrogen content structures that we investigated upon decompression. Na_3_WH_9_ can be synthesized above 7.8 GPa after laser heating, featuring 18-electron [WH_9_]^3–^ homoleptic units. In the Na–Re–H system, Na_3_ReH_8_ was synthesized at 32.5 and 10.1 GPa, with [ReH_8_]^3–^ units. Upon decompression from about 40 GPa, these structures undergo analogous phase transitions: II′(distorted fcc) → II (Heusler fcc)→ I (distorted hcp). The specific distortion scenarios and transition pressures depend on the transition metal. Experiments and theoretical calculations indicate that these ternary compounds are stable to pressures below 1 GPa, making them good candidates for hydrogen storage materials, however, Na_3_WH_9_ and Na_3_ReH_8_ are electrical insulators with wide bandgaps. The thermodynamic stability of the different observed polymorphs was studied via DFT and MD calculations, showing excellent compatibility with the experimentally reported structural transformations.

## Supporting Information Available

Description of the experimental and computational details, (ii) additional XRD and Raman data from different samples, (iii) results on MD calculations, (iv) calculated pressure evolution of the band gap of Na_3_WH_9_ and (v) DFT calculated atomic positions for different phases (PDF)

## Notes

For the purpose of open access, the authors have applied a Creative Commons Attribution (CC BY) license to any Author Accepted Manuscript version arising from this submission.
