# Supplementary material for: Synthesis of Na3WH9 and Na3ReH8 Ternary Hydrides at High Pressures
Source: Inorg Chem. 2024 Oct 31;63(45):21734–41. doi: 10.1021/acs.inorgchem.4c02691 (PMC11558676; doi:10.1021/acs.inorgchem.4c02691)
Supplement: Supplementary file 1 — ic4c02691_si_001.pdf [file ic4c02691_si_001.pdf]

Supporting Information Synthesis of Na_3WH_9 and Na_3ReH_8 Ternary Hydrides at High Pressures

Tomas Marqueño,[†] Israel Osmond,[†] Mikhail A. Kuzovnikov,[†] Hannah A. Shuttleworth,[†] Samuel Gallego-Parra,[‡] Eugene Gregoryanz,[†,¶,§] Andreas Hermann,[†] Ross T. Howie,[†,§] and Miriam Peña-Alvarez[*,†]

†*Center for Science at Extreme Conditions (CSEC) and the School of Physics and Astronomy, The University of Edinburgh, Peter Guthrie Tait Road, Edinburgh EH3 9FD, United Kingdom*

‡*European Synchrotron Radiation Facility (ESRF), Grenoble 38000, France*

¶*Key Laboratory of Materials Physics, Institute of Solid State Physics, HFIPS, Chinese Academy of Sciences, Hefei 230031, China*

§*Center for High Pressure Science and Technology Advanced Research (HPSTAR), Shanghai 201203, China*

E-mail: miriam.pena.alvarez@ed.ac.uk

Experimental Details

The Na-W-H and Na-Re-H systems were studied under high pressure using diamond anvil cells (DACs), equipped with diamonds with culet sizes of 200 μm. Rhenium gaskets were preindented, laser drilled and placed between the diamonds. Sodium monohydride (Sigma Aldrich, 95% dry) was loaded into the DAC chambers inside of a glovebox under an argon

atmosphere to prevent the oxidation of NaH, together with small grains of gold, used as pressure calibrant.[1] In the case of the Na-W-H system, a grain of tungsten (W) was also placed into the gasket hole. DACs were closed in a hydrogen atmosphere (99.9999%) at 2000 bar. The samples were laser heated using an infrared neodymium-glass laser with $\lambda = 1058$ nm. We used the grain of W and the Re gasket as radiation couplers for the Na-W-H and Na-Re-H systems, respectively.

The samples were characterized by means of both Powder X-ray diffraction (PXRD) and Raman scattering. PXRD measurements were performed at the P02.0 ($\lambda = 0.2904$, 0.2914 Å) and ID15B ($\lambda = 0.4100$ Å) beamlines at DESY (PETRA III) and the ESRF synchrotron facilities, respectively.[2,3] In the former beamline, the beam was focused down to a spot size of $2 \times 2\ \mu m^2$ and the data was acquired using a Perkin Elmer XRD 1621 (CsI bonded amorphous silicon detector). At the ID15B beamline (ESRF), the beam was focused down to a spot size of $5 \times 5\ \mu m^2$. A large area EIGER2 X 9M CdTe (340×370 mm) flat panel detector was used to measure the diffracted patterns. The integration of the PXRD patterns was performed using DIOPTAS.[4] The analysis and refinements of the obtained PXRD patterns were carried out using the PowderCell and GSAS-II software packages.[5,6]

Raman spectra of the samples were acquired using an custom-built microfocused Raman setup, equipped with a liquid nitrogen cooled detector. In this system we used an green argon laser with an excitation wavelength of $\lambda = 514.47$ nm. In order to measure Raman spectra in a wide range of frequencies, a grating of 500 lines/mm was used. Pressure was monitored using both diamond edge[7] and the pressure calibration of the H_2 highest frequency vibron.[8]

Simulation Details

Density functional theory (DFT) calculations were performed for structure optimizations, molecular dynamics (MD) simulations, and phonon calculations, using the Vienna ab initio simulation package VASP[9] together with PAW pseudopotentials[10] that included 7/13/14

valence electrons for Na/Re/W, respectively. Electronic exchange-correlation effects were described with the Perdew-Burke-Ernzerhof (PBE) functional.[11] Geometry optimisations used the "hard" PAW dataset for H, a plane wave cutoff energy of $E_c = 800$ eV, and regular Brillouin zone sampling grids with linear density $20/\text{Å}^{-1}$; while MD simulations use the "standard" PAW dataset for H, a plane wave cutoff energy of $E_c = 300$ eV, and the Baldereschi point $(1/4, 1/4, 1/4)$ in the Brillouin zone. Geometries were optimised until residual forces were below 1 meV/Å, and residual stresses below 0.1 GPa.

Experiments cannot constrain hydrogen composition or positions, and various approaches were taken to construct full crystal structures. For Na_3WH_9 and Na_3ReH_8, the heavy atom unit cells were populated initially with sensible hydrogen content (n=9/10 for W; n=7/8 for Re) and it quickly became clear that the compositions presented here are energetically most favourable. Phonon calculations were then used to detect dynamical instabilities, and these were followed until resulting structures (in lower symmetries, and larger unit cells) were dynamically stable. Ultimately, this reduced the high-pressure symmetries from $Immm$ to $P2_1/c/Pca2_1$, and the low-pressure symmetries from $P6_3/mmc$ to $P6_3/m/Pbca$.

MD simulations were run within the NPT ensemble, realised using the Parrinello-Rahman method,[12,13] at $T = 300$ K and a series of pressure points. The timestep in the MD was 0.5 fs. (2,2,2) supercells of the conventional fcc lattice were used to model the transition to a Heusler-type fcc structure; the equilibration of the supercell lattice lengths is therefore a measure of the transition to a quasi-cubic phase.

Phonon calculations used the finite displacement method and suitable supercells to determine the real space force constant matrix, as implemented in the phonopy package.[14,15] Symmetry analyses on the zone-centered eigenmodes were used to determine the Raman-active modes; Raman intensity calculations using the phonopySpectroscopy package[16] were not successful due to persistent errors in the VASP density functional perturbation theory (DFPT) calculations.

Formation enthalpies of the ternary hydrides were evaluated against pure W/Re in their

bcc/hcp phases; NaH in both B1 (NaCl-type) and B2 (CsCl-type) structures; and H_2 in both $P6_3/m$ (model for phase I) and $C2/c$ (model for phase III) structures.

Refined structures (main text)

Table S1: Refined unit-cell parameters for Na_3WH_9-II' ($P2_1/c$) at $P = 23.5$ GPa (**Fig. 1(b)**, $R_{wp} = 29.7$ %). Refined lattice parameters are $a = 4.976(2)$ Å, $b = 10.488(3)$ Å, $c = 8.625(2)$ Å, $\beta = 54.1(3)°$. Preferred orientations were accounted using the March-Dollase model (coef. = 0.59, axis = (1 2 -1)).

**Atom**	**Wyckoff position**	x	y	z	U_{iso}
W	4e	0.991(5)	0.135(9)	0.247(5)	0.022(2)
Na	4e	0.26(2)	0.91(3)	0.02(2)	0.01 (fixed)
Na	4e	0.81(2)	0.60(3)	0.99(2)	0.01 (fixed)
Na	4e	0.50(3)	0.853(5)	0.25(3)	0.01 (fixed)

Table S2: Refined unit-cell parameters for Na_3WH_9-II (fcc) at $P = 6.4$ GPa (**Fig. 1(c)**, $R_{wp} = 33.5$ %). Refined lattice parameter is $a = 7.660(2)$ Å. Preferred orientations were accounted using the March-Dollase model (coef. = 0.58, axis = (111)).

**Atom**	**Wyckoff position**	x	y	z	U_{iso}
W	4a	0	0	0	0.026(2)
Na	8c	1/4	1/4	1/4	0.076(9)
Na	4b	1/2	1/2	1/2	0.06(2)

Table S3: Refined unit-cell parameters for Na_3WH_9-I ($P6_3/m$) at $P < 2$ GPa (**Fig. 1(d)**, $R_{wp} = 37.7$ %). Refined lattice parameters are $a = b = 5.590(2)$ Å, $c = 10.059(2)$ Å.

**Atom**	**Wyckoff position**	x	y	z	U_{iso}
W	2d	2/3	1/3	1/4	0.031(2)
Na	2a	0	0	1/4	0.04(2)
Na	4f	1/3	2/3	0.421(4)	0.06(1)

Table S4: Refined unit-cell parameters for Na_3ReH_8-II' ($Pca2_1$) at $P = 32.5$ GPa (**Fig. 2(b)**, $R_{wp} = 31.2$ %). Lattice parameters are $a = 9.711(1)$ Å, $b = 6.898(1)$ Å, $c = 9.607(2)$ Å.

**Atom**	**Wyckoff position**	x	y	z	U_{iso}
Re	4a	0.245(2)	0.497(2)	0.13(1)	0.002(5)
Re	4a	0.01(2)	0.015(8)	0.87(1)	0.007(4)
Na	4a	0.767(8)	0.18(1)	0.85(1)	0.01 (fixed)
Na	4a	0.25(1)	0.23(2)	0.92(2)	0.01 (fixed)
Na	4a	0.98(2)	0.19(2)	0.13(1)	0.01 (fixed)
Na	4a	0.50(1)	0.25(1)	0.10(1)	0.01 (fixed)
Na	4a	0.027(7)	0.49(1)	0.85(2)	0.01 (fixed)
Na	4a	0.25(1)	0.06(1)	0.12(2)	0.01 (fixed)

Table S5: Refined unit-cell parameters for Na_3ReH_8-II (fcc) at $P = 14.6$ GPa (**Fig. 2(c)**, $R_{wp} = 33.0$ %). Refined lattice parameters are $a = b = c = 7.2480(5)$ Å.

**Atom**	**Wyckoff position**	x	y	z	U_{iso}
Re	4a	0	0	0	0.024(1)
Na	8c	1/4	1/4	1/4	0.01 (fixed)
Na	4b	1/2	1/2	1/2	0.01 (fixed)

Table S6: Refined unit-cell parameters for Na_3ReH_8-I ($Pbca$) at $P = 1.8$ GPa (**Fig. 2(d)**, $R_{wp} = 32.6$ %). Lattice parameters are $a = 9.511(7)$ Å, $b = 9.393(2)$ Å, $c = 11.002(9)$ Å.

**Atom**	**Wyckoff position**	x	y	z	U_{iso}
Re	8c	0.5897(22)	0.7434(8)	0.377(1)	0.011(1)
Na	8c	0.23(2)	0.749(7)	0.373(9)	0.03(3)
Na	8c	0.43(2)	0.622(6)	0.15(1)	0.03(4)
Na	8c	0.45(1)	0.558(7)	0.653(7)	0.03(3)

Supplementary Figures

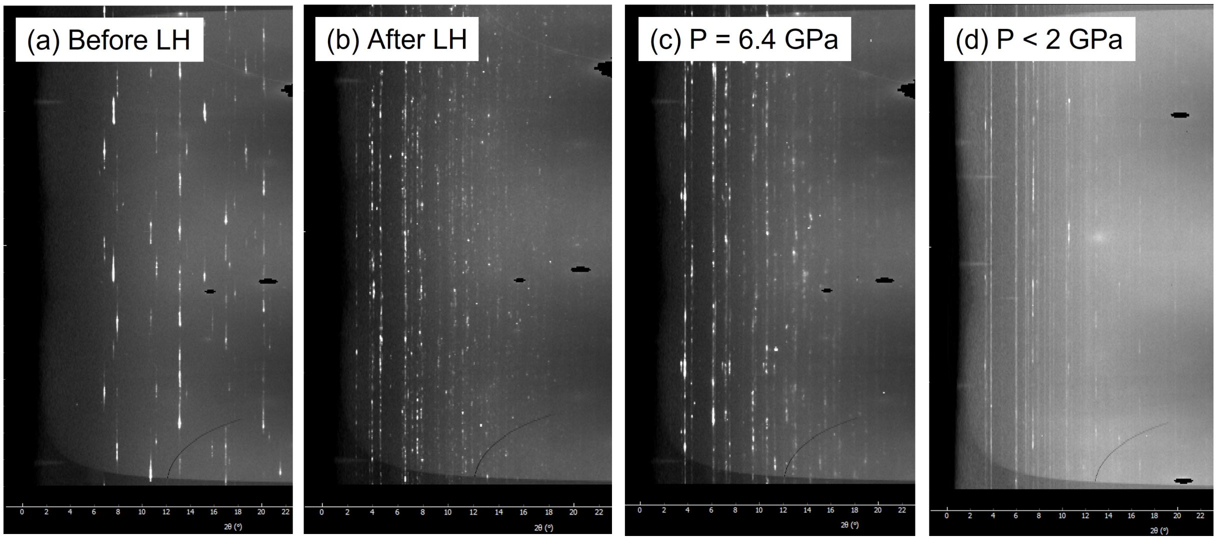

Fig. S1: XRD detector images of the sample taken (a) before laser heating, (b) after laser heating (P = 23.5 GPa), and (c) at 6.4 GPa after decompression, for the first set of experiments ($\lambda = 0.2904$ Å); (d) shows the image plate of the sample at pressures below 2 GPa, for the second run of experiments ($\lambda = 0.2914$ Å). The image in this case corresponds to the low pressure phase of Na_3WH_9-I.

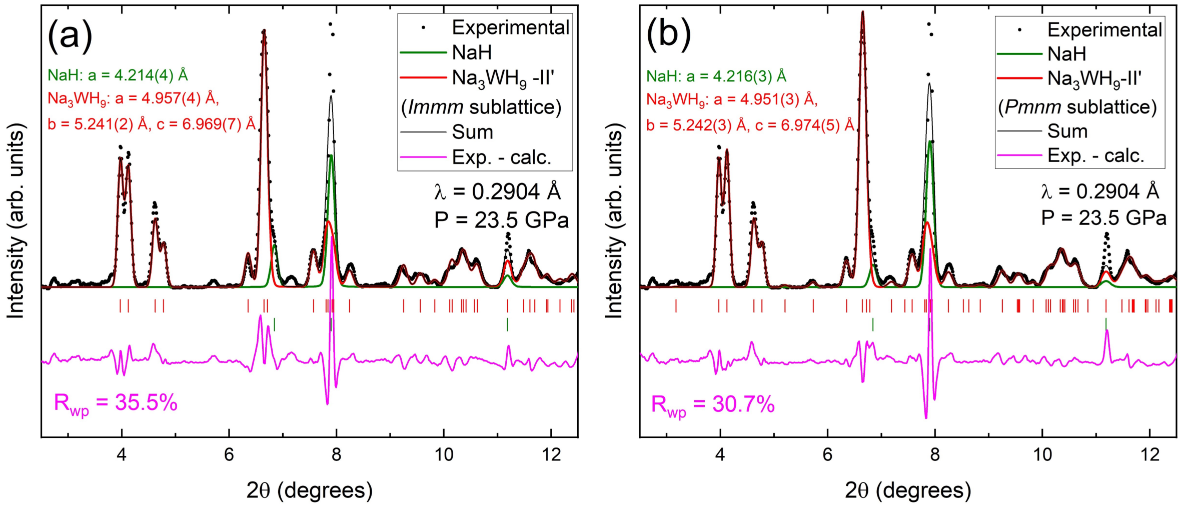

Fig. S2: Rietveld refinements for the XRD pattern shown in Fig. 1(b) using different Na-W sublattices for Na_3WH_9-II' i.e. space groups (a) *Immm* and (b) *Pmnm*. Black dots are the experimental data while red and green solid lines represent the Rietveld refinements for the Na_3WH_9-II' and NaH-I phases, respectively. The difference between the refinement and the observed pattern is depicted as magenta lines. Refined parameters are given in Tables S7 and S8 (below).

Table S7: Refined unit-cell parameters for Na_3WH_9-II' (*Immm* sublattice) at $P = 23.5$ GPa (**Fig. S2(a)**, $R_{wp} = 35.5$ %). Refined lattice parameters are $a = 4.957(4)$ Å, $b = 5.241(2)$ Å, $c = 6.969(7)$ Å. Preferred orientations were accounted using the March-Dollase model (coef. = 0.645, axis = (1 1 2)).

**Atom**	**Wyckoff position**	x	y	z	U_{iso}
W	2a	0	0	0	0.039(5)
Na	2c	1/2	1/2	0	0.01 (fixed)
Na	4j	1/2	0	0.75(1)	0.01 (fixed)

Table S8: Refined unit-cell parameters for Na_3WH_9-II' ($Pmnm$ sublattice, origin at -1) at $P = 23.5$ GPa (**Fig. S2(b)**, $R_{wp} = 30.7$ %). Refined lattice parameters are $a = 4.951(3)$ Å, $b = 5.242(3)$ Å, $c = 6.974(5)$ Å. Preferred orientations were accounted using the March-Dollase model (coef. = 0.599, axis = (1 1 2)).

**Atom**	**Wyckoff position**	x	y	z	U_{iso}
W	2a	1/4	0.271(2)	1/4	0.019(3)
Na	2b	3/4	0.72(2)	1/4	0.01 (fixed)
Na	4f	1/4	0.80(3)	0.01(2)	0.01 (fixed)

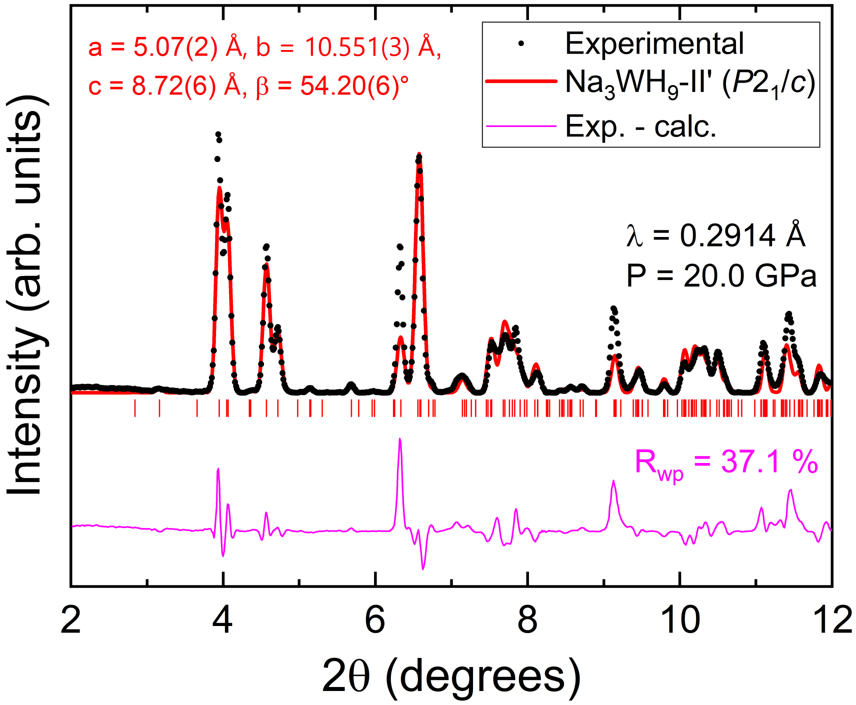

Fig. S3: XRD pattern collected after laser heating the sample at 20 GPa in the second set of XRD experiments for the Na-W-H system ($\lambda = 0.2914$ Å). Experimental data is depicted as black dots. The red solid line represents the Rietveld refinement for the Na_3WH_9-II' phase. Magenta lines represent the difference between the experimental and calculated pattern. Bragg reflections are indicated with vertical ticks. Refined parameters are shown in Table S9.

Table S9: Refined unit-cell parameters for Na_3WH_9-II' ($P2_1/c$) at $P = 20.0$ GPa (**Fig. S3**, $R_{wp} = 37.1$ %). Refined lattice parameters are $a = 5.07(2)$ Å, $b = 10.551(3)$ Å, $c = 8.72(6)$ Å, $\beta = 54.20(6)°$.

**Atom**	**Wyckoff position**	x	y	z	U_{iso}
W	4e	0.007(4)	0.1385(5)	0.250(2)	0.02(3)
Na	4e	0.21(2)	0.903(5)	0.941(9)	0.01 (fixed)
Na	4e	0.81(2)	0.613(4)	0.983(9)	0.01 (fixed)
Na	4e	0.57(3)	0.857(4)	0.23(1)	0.01 (fixed)

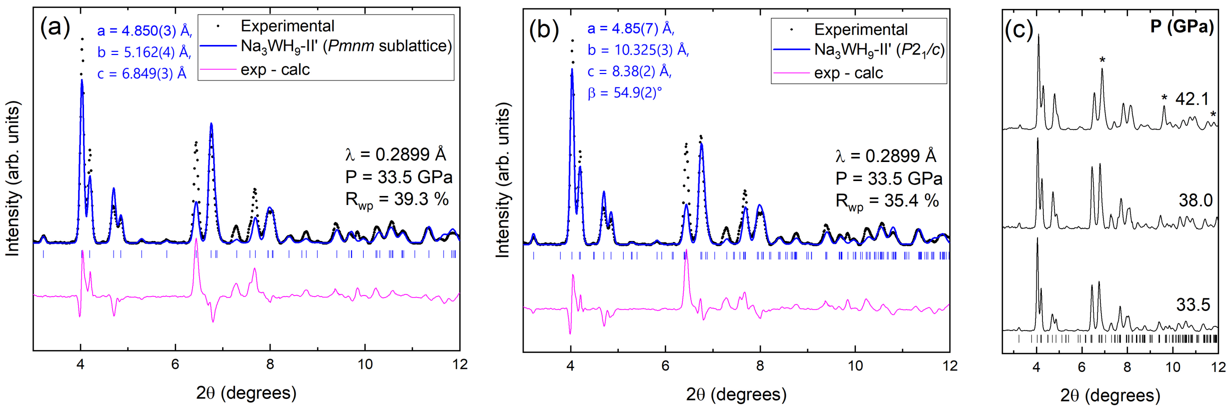

Fig. S4: (a) and (b) XRD patterns of the sample obtained after laser heating NaH, W and H_2 at 33.5 GPa. Experimental values are depicted as circles. The corresponding Rietveld refinement of the Na_3WH_9-II' structure is shown with a blue solid line. The space groups considered for the refinements shown in (a) and (b) are $Pmnm$ (initial guess) and $P2_1/c$ (obtained from DFT calculations), respectively. The position of the Bragg reflections is indicated with blue vertical ticks. The magenta line represents the difference between the experimental data and the refinement. The refined parameters are summarized in Table S10 and S11 for (a) and (b), respectively (below). (c) Selected XRD patterns of Na_3WH_9-II' at different pressures over 33.5GPa upon compression. Vertical ticks represent the position of the Bragg reflections of this compound at 33.5 GPa. Reflections associated to NaH-II are indicated with asterisks.

Table S10: Refined unit-cell parameters for Na_3WH_9-II' ($Pmnm$ sublattice) at $P = 33.5$ GPa (**Fig. S4**, $R_{wp} = 39.3$ %). Refined lattice parameters are $a = 4.850(3)$ Å, $b = 5.162(4)$ Å, $c = 6.849(3)$ Å. Preferred orientations were accounted using the March-Dollase model (coef. = 0.74, axis = (0 1 1)).

**Atom**	**Wyckoff position**	x	y	z	U_{iso}
W	2a	1/4	0.281(3)	1/4	0.035(3)
Na	2b	3/4	0.67(3)	1/4	0.01 (fixed)
Na	4f	1/4	0.77(2)	0.02(1)	0.01 (fixed)

Table S11: Refined unit-cell parameters for Na_3WH_9-II' ($P2_1/c$) at $P = 33.5$ GPa (**Fig. S4**, $R_{wp} = 35.4$ %). Refined lattice parameters are $a = 4.85(7)$ Å, $b = 10.325(3)$ Å, $c = 8.38(2)$ Å, $\beta = 54.9(2)°$. Preferred orientations were accounted using the March-Dollase model (coef. = 0.671, axis = (0 2 1)).

**Atom**	**Wyckoff position**	x	y	z	U_{iso}
W	4e	0.996(9)	0.1402(7)	0.244(4)	0.035(3)
Na	4e	0.18(5)	0.875(9)	0.08(1)	0.01 (fixed)
Na	4e	0.78(5)	0.615(7)	0.97(2)	0.01 (fixed)
Na	4e	0.52(5)	0.847(4)	0.246(2)	0.01 (fixed)

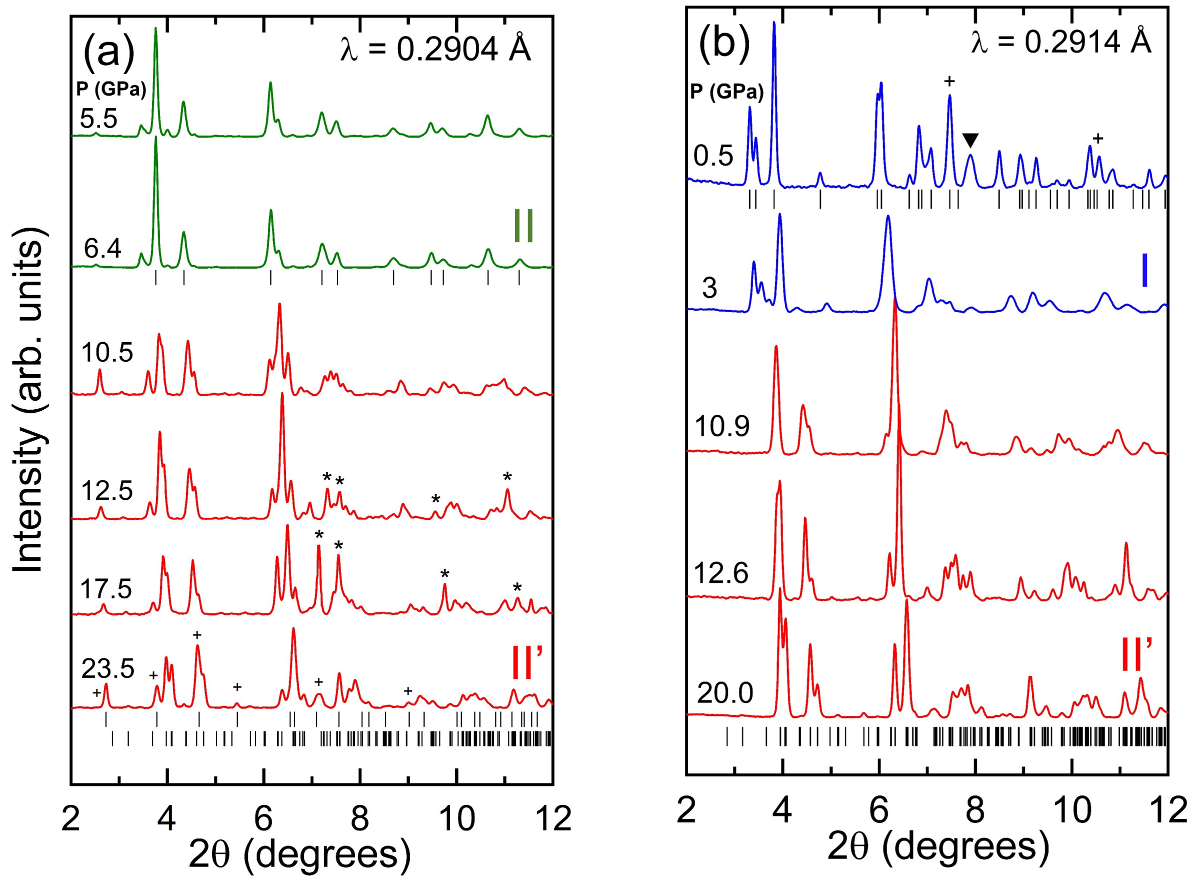

Fig. S5: Selected XRD patterns of Na-W-H compounds at different pressures, collected upon decompression for the (a) first and (b) second set of diffraction experiments. The patterns in (a) also show some extra Bragg reflections (plus symbols) that can be associated with a phase with primitive simple hexagonal lattice of W atoms ($P6_3/mmm$, $a = 5.079(3)$ Å, $c = 12.223(5)$ Å) that decomposes upon decompression. Asterisks in (a) indicate reflections associated to WH; Plus and triangle symbols in (b) denote reflections corresponding to tungsten and rhenium hydride, respectively.

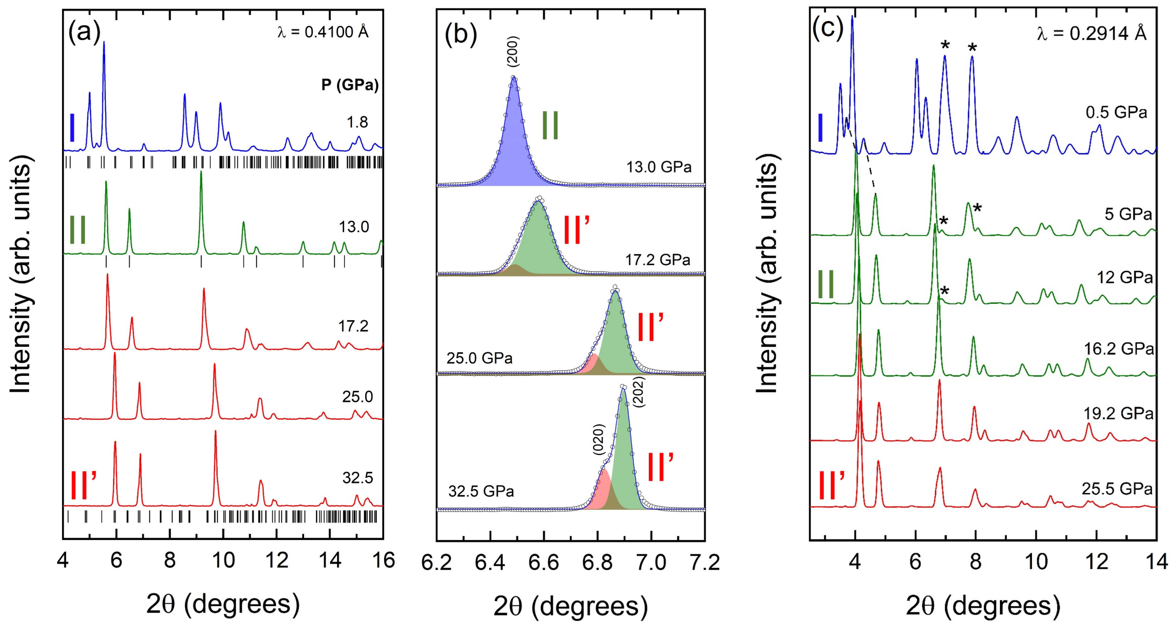

Fig. S6: Selected powder XRD of Na_3ReH_8 at different pressures upon decompression. (a) show the patterns collected during a first set of experiments (λ = 0.41 Å); (b) shows a detailed section (2θ = 6.2 - 7.2°) of the patterns in (a) between 32.5 and 13.0 GPa, showing how the (020) and (202) reflections of Na_3ReH_8-II' (assuming space group $Pca2_1$) merge and become the (200) reflection of fcc Na_3ReH_8-II. The patterns collected during a second run are shown in (c). Asterisk denote reflections associated to rhenium hydride.

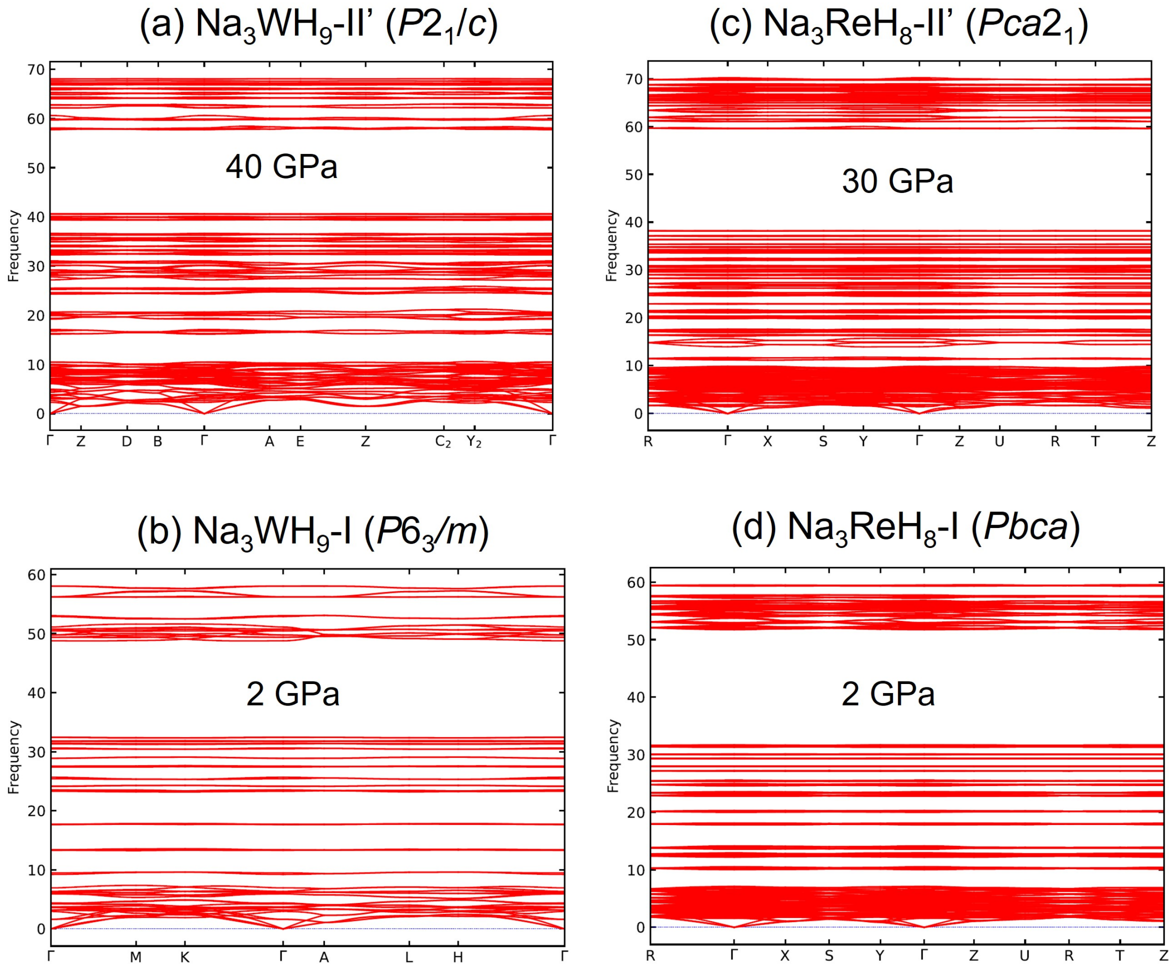

Fig. S7: Calculated phonon bands for (a) Na_3WH_9-II' ($P2_1/c$) (b) Na_3WH_9-I ($P6_3/m$), (c) Na_3ReH4_8-II' ($Pca2_1$) (d) Na_3ReH_8-I ($Pbca$). Pressures are indicated within the figure. All frequencies are in THz.

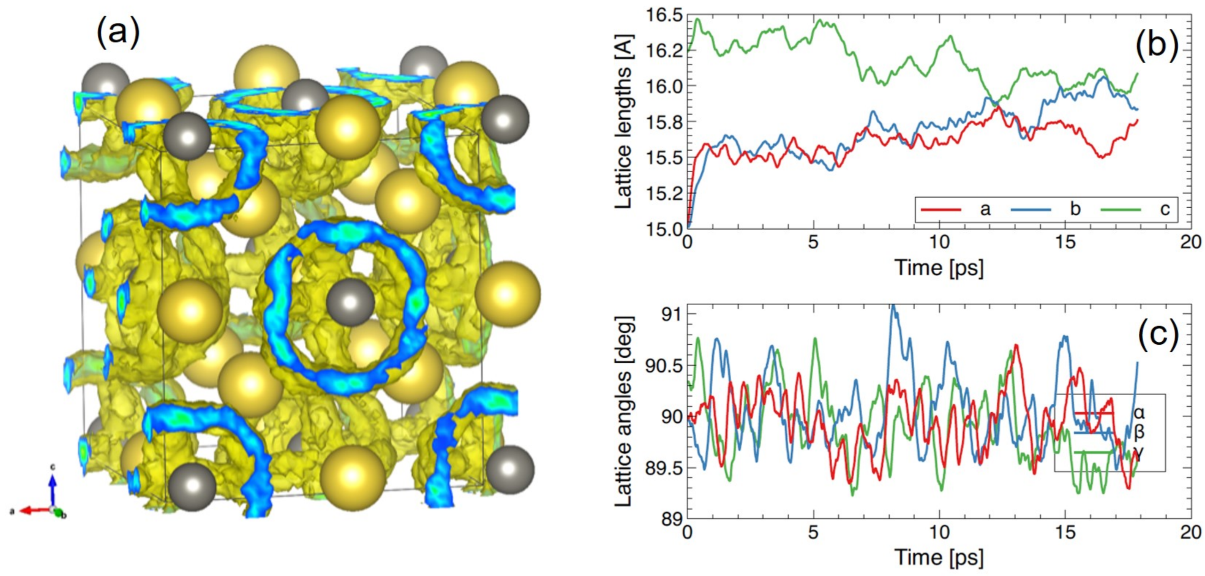

Fig. S8: DFT-MD simulations of the Heusler fcc-like structure of Na_3WH_9-II at 4 GPa. (a) Time-averaged quasi-cubic unit cell and Na/W positions (yellow and grey spheres, respectively). Yellow isosurfaces represent H atom probability distributions, which form concentric spheres around W atoms due to the rotational state of the WH_9 anions. (b) and (c) Time evolution of the MD supercell's lattice lengths and angles, respectively, showing that the structure transforms from an orthorhombic to a cubic phase after about 12 ps simulation time.

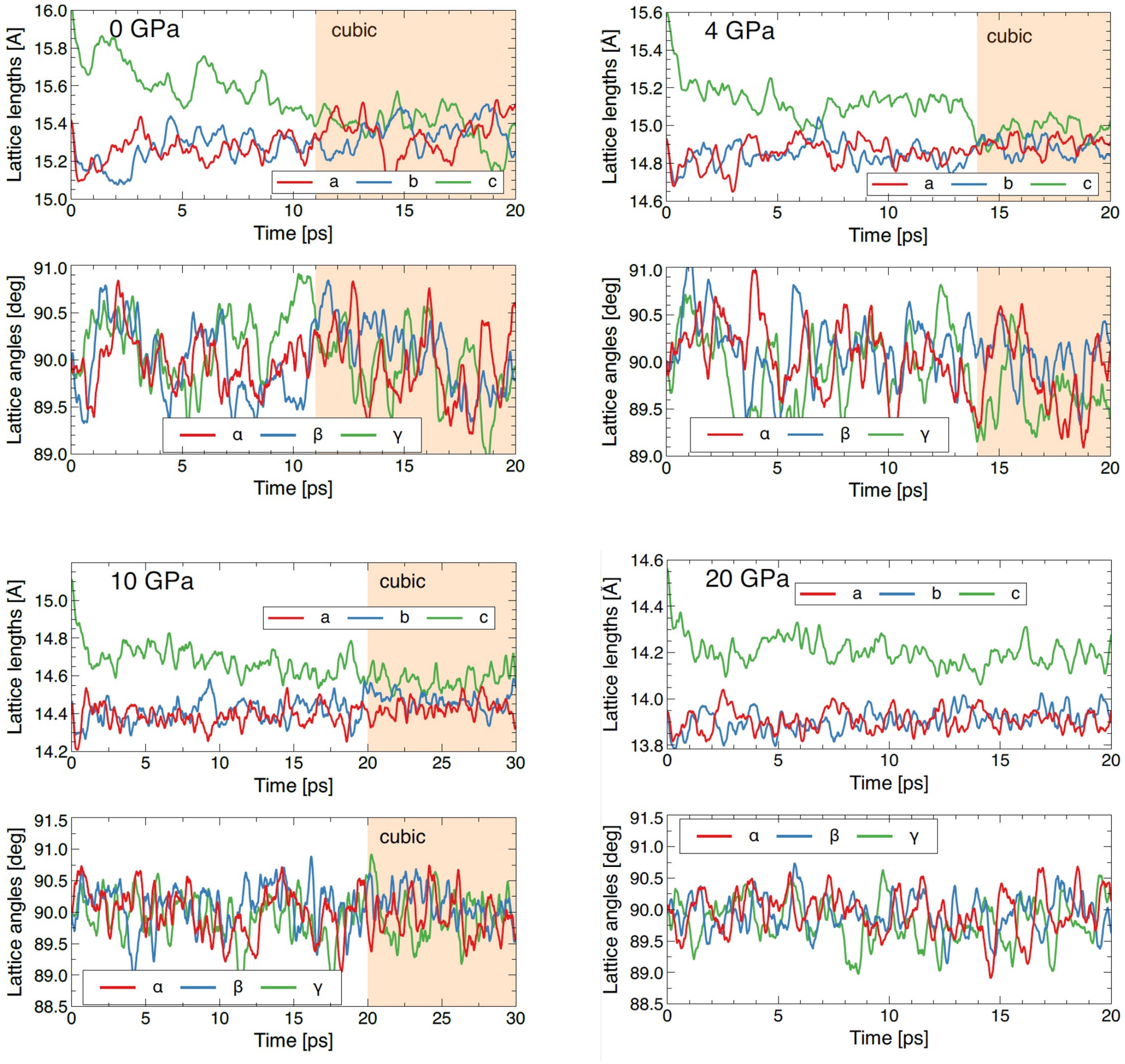

Fig. S9: Time evolution for the lattice lengths and angles of Heusler-type Na_3ReH_8-II' at 0, 4, 10 and 20 GPa, from DFT-MD simulations. The approximate onset of the cubic phase, judged by lattice length equilibration, is marked. Hydrogen mobility is reduced with pressure, so the transformation to the cubic phase becomes slower at higher pressure. At 20 GPa the lattice lengths do not converge to the same average value within 20 ps, which suggests that at this pressure the fcc structure cannot be achieved.

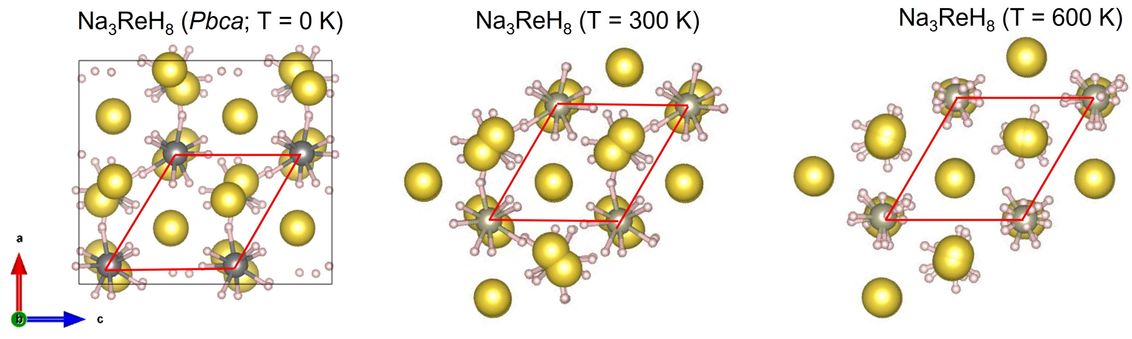

Fig. S10: Comparison of the simulated low-pressure crystal structure of Na_3ReH_8-I at different temperatures (0, 300 and 600 K). The snapshots at 300 and 600 K show how the hexagonal structure is achieved as a consequence of dynamic disorder upon increasing temperatures. Na, Re and H atoms are represented with yellow, grey and white spheres, respectively. The *hcp* lattice formed by Re atoms is highlighted with red lines.

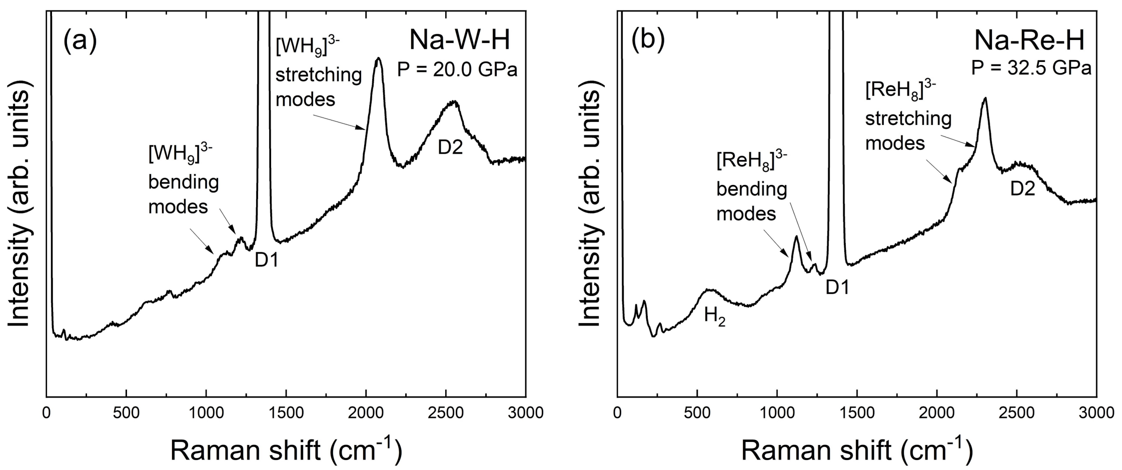

Fig. S11: Raman spectra of the (a) Na_3WH_9-II' and (b) Na_3ReH_8-II' samples that were previously measured using XRD, whose corresponding diffraction patterns are shown in Figs. 1 (b) and 2 (b), respectively.

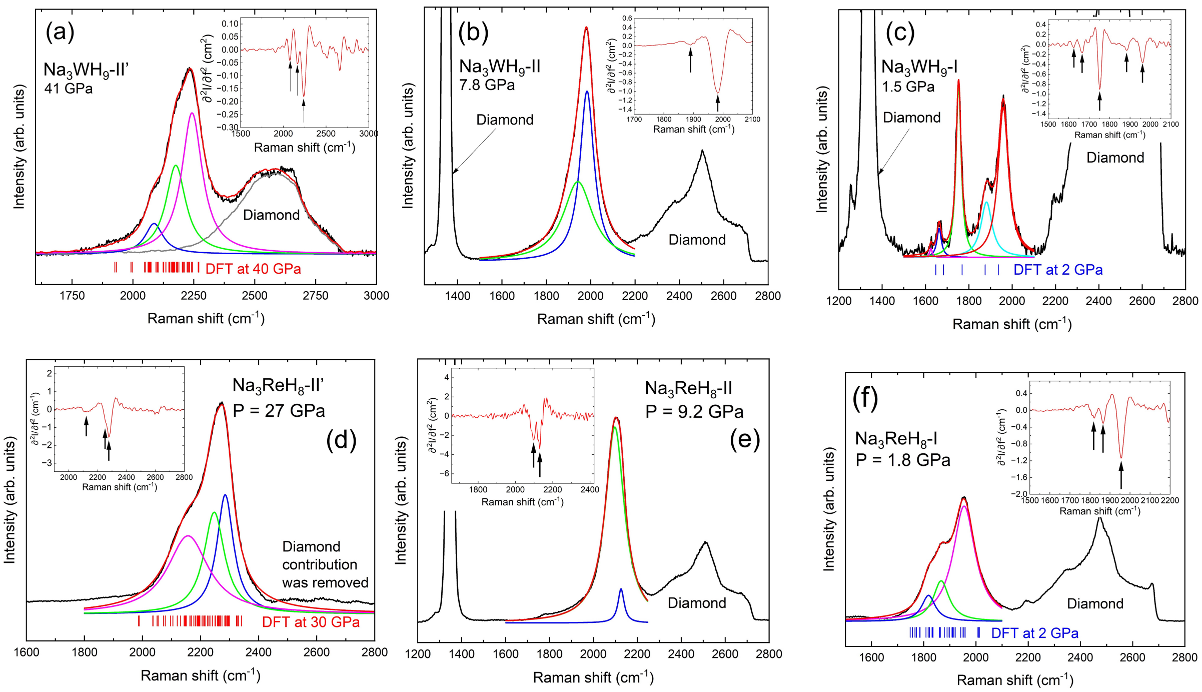

Fig. S12: Examples of peak fittings for the stretching modes band for each phase of Na_3WH_9 [(a) II', (b) II, (c) I] and Na_3ReH_8 [(d) II', (e) II, (f) I]. The inset shows the second derivative analysis of the corresponding Raman bands. Arrows identify the position of the minima.

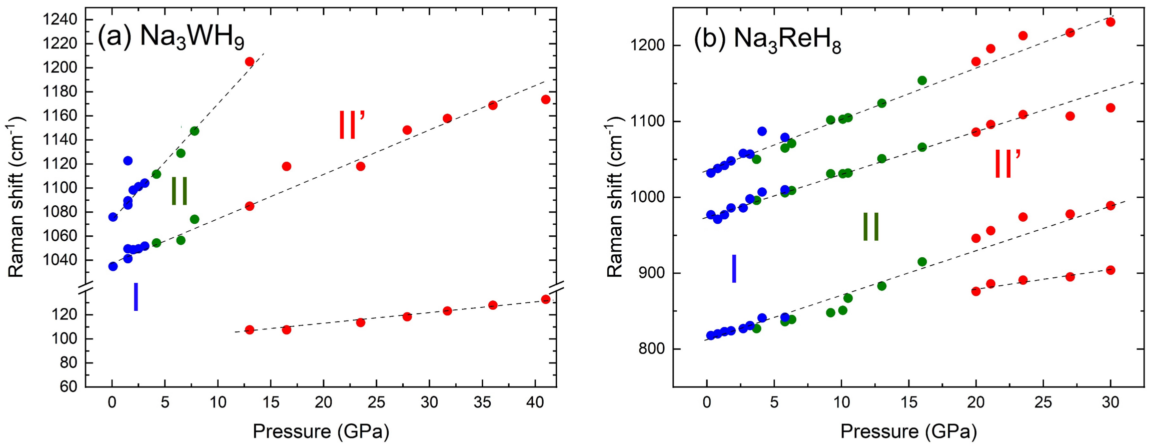

Fig. S13: Frequency dependence with pressure of Raman bands associated with bending and lattice modes of (a) Na_3WH_9 and (b) Na_3ReH_8. Red, green and blue symbols correspond to the II', II and I phases of each compound, respectively. Dashed lines are a guide to the eye.

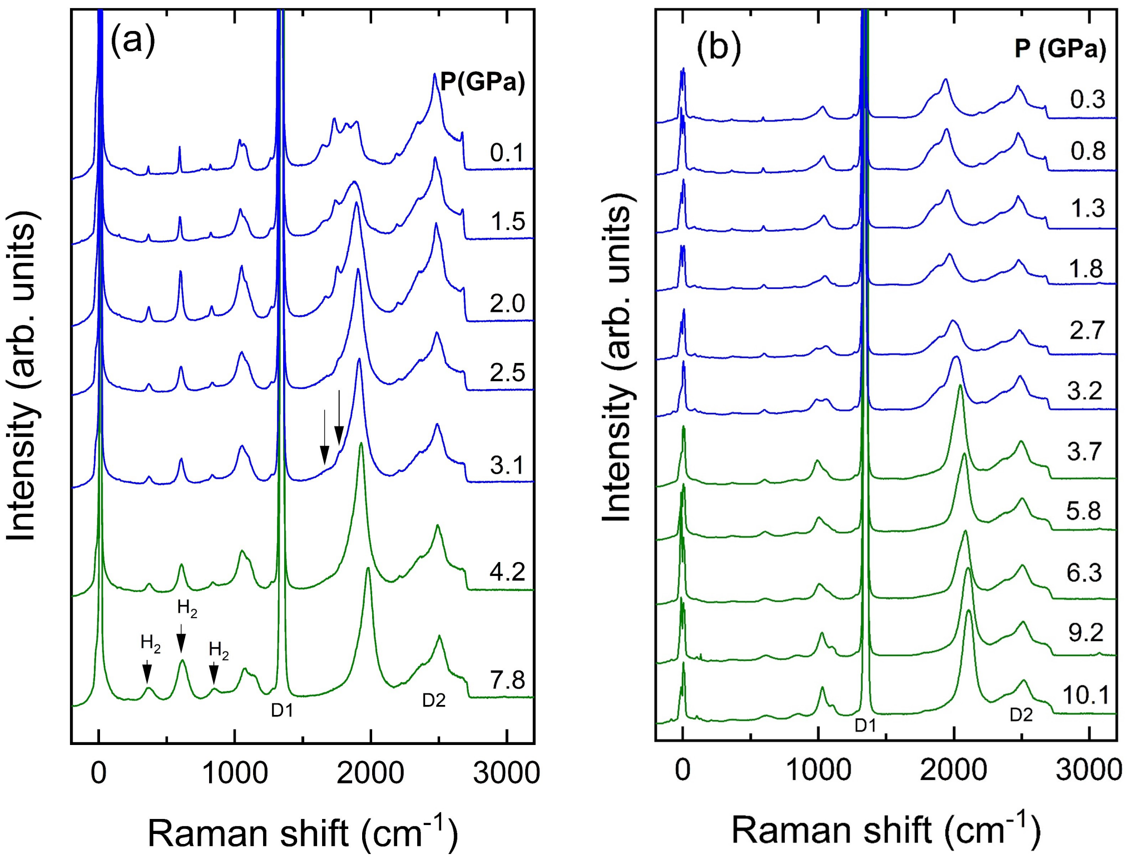

Fig. S14: Raman spectra of (a) Na_3WH_9 and (b) Na_3ReH_8 upon decompression. The Raman spectra where the II and I phases are dominant are depicted with green and blue lines, respectively. Peaks denoted as "H_2 correspond to hydrogen rotons. Arrows indicate new peaks emerging during the phase transition.

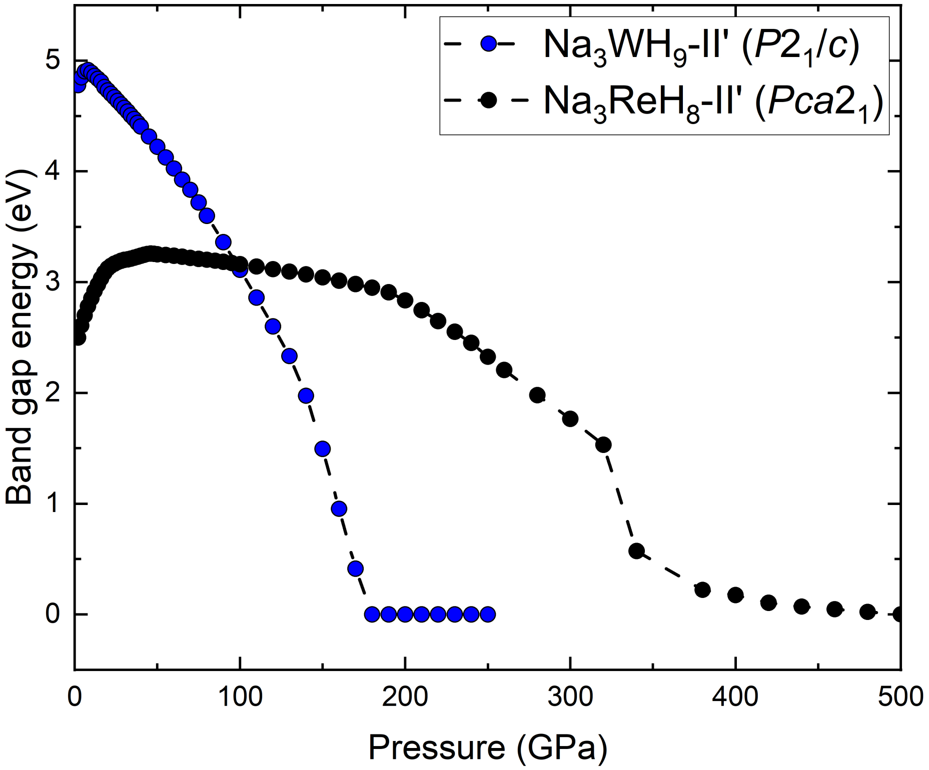

Fig. S15: Calculated pressure evolution of the electronic band gaps of Na_3WH_9-II' ($P2_1/c$) and Na_3ReH_8-II' ($Pca2_1$).

Calculated structures

Table S12: Calculated crystal structure for Na_3WH_9-II' ($P2_1/c$) at 20 GPa. Lattice parameters are $a = 5.0393$ Å $b = 10.4346$ Å, $c = 8.7858$ Å, $\beta = 53.1213°$.

**Atom**	**Wyckoff position**	x	y	z
W	4e	0.00110	0.14026	0.24090
Na	4e	0.25950	0.89445	0.98339
Na	4e	0.77809	0.59763	0.97969
Na	4e	0.50779	0.84976	0.24128
H	4e	0.11925	0.70245	0.71727
H	4e	0.36757	0.66386	0.22119
H	4e	0.16136	0.49448	0.27654
H	4e	0.95126	0.70767	0.45030
H	4e	0.30802	0.90165	0.52092
H	4e	0.78422	0.52453	0.21908
H	4e	0.68770	0.92492	0.97095
H	4e	0.93419	0.71019	0.11040
H	4e	0.64869	0.76617	0.88139

Table S13: Calculated crystal structure for Na_3ReH_8-II' ($Pca2_1$) at 30 GPa. Lattice parameters are $a = 9.7128$ Å, $b = 6.9615$ Å, $c = 9.629$ Å.

**Atom**	**Wyckoff position**	x	y	z
Re	4a	0.25592	0.49540	0.13513
Re	4a	0.02198	0.01075	0.86489
Na	4a	0.76383	0.23640	0.89292
Na	4a	0.30032	0.26237	0.88067
Na	4a	0.98871	0.23520	0.12302
Na	4a	0.50675	0.26559	0.10418
Na	4a	0.02631	0.46226	0.87242
Na	4a	0.24944	0.03126	0.12473
H	4a	0.18854	0.01256	0.83157
H	4a	0.10233	0.47217	0.21390
H	4a	0.85004	0.42651	0.51016
H	4a	0.31178	0.30805	0.55499
H	4a	0.89544	0.05823	0.50756
H	4a	0.41032	0.20167	0.43946
H	4a	0.08224	0.52096	0.67935
H	4a	0.11590	0.04473	0.27089
H	4a	0.18477	0.29037	0.69952
H	4a	0.74437	0.25959	0.64923
H	4a	0.96850	0.23475	0.35361
H	4a	0.55226	0.20968	0.30676
H	4a	0.13786	0.50889	0.49530
H	4a	0.10509	0.99485	0.48659
H	4a	0.05941	0.02097	0.69897
H	4a	0.77036	0.48086	0.30417

Table S14: Calculated crystal structure for Na_3WH_9-I ($P6_3/m$) at 2 GPa. Lattice parameters are $a = b = 5.4616$ Å, $c = 9.8188$ Å.

**Atom**	**Wyckoff position**	x	y	z	**Symmetry**
W	2d	2/3	1/3	1/4	$-6..$
Na	2a	0	0	1/4	$-6..$
Na	4f	1/3	2/3	0.42189	3..
H	6h	0.02244	0.43668	1/4	$m..$
H	12i	0.74322	0.15204	0.12093	1

Table S15: Calculated crystal structures for Na_3ReH_8-I ($Pbca$) at 2 GPa. Lattice parameters are $a = 9.5631$ Å, $b = 9.3177$ Å, $c = 10.8539$ Å.

**Atom**	**Wyckoff position**	x	y	z
Re	8c	0.58539	0.74532	0.38136
Na	8c	0.25256	0.71630	0.36357
Na	8c	0.37742	0.57058	0.08994
Na	8c	0.45908	0.58319	0.64143
H	8c	0.75600	0.78834	0.40786
H	8c	0.85282	0.13094	0.49278
H	8c	0.54756	0.13827	0.06392
H	8c	0.55173	0.13533	0.62842
H	8c	0.33026	0.38978	0.69819
H	8c	0.00892	0.29822	0.75244
H	8c	0.58039	0.64877	0.01126
H	8c	0.85585	0.37143	0.27593

References

(1) Dorogokupets, P.; Dewaele, A. Equations of state of MgO, Au, Pt, NaCl-B1, and NaCl-B2: Internally consistent high-temperature pressure scales. *High Press. Res.* **2007**, *27*, 431–446.

(2) Liermann, H.-P.; Konôpková, Z.; Morgenroth, W.; Glazyrin, K.; Bednarčik, J.; McBride, E.; Petitgirard, S.; Delitz, J.; Wendt, M.; Bican, Y.; others The extreme conditions beamline P02. 2 and the extreme conditions science infrastructure at PETRA III. *J. Synchrotron Radiat.* **2015**, *22*, 908–924.

(3) Comboni, D.; Hanfland, M.; Garbarino, G. ID15b, Crystallogr. Earth Sci. **2022**,

(4) Prescher, C.; Prakapenka, V. B. DIOPTAS: a program for reduction of two-dimensional X-ray diffraction data and data exploration. *High Press. Res.* **2015**, *35*, 223–230.

(5) Kraus, W.; Nolze, G. POWDER CELL–a program for the representation and manipulation of crystal structures and calculation of the resulting X-ray powder patterns. *J. Appl. Crystallogr.* **1996**, *29*, 301–303.

(6) Toby, B. H.; Von Dreele, R. B. GSAS-II: the genesis of a modern open-source all purpose crystallography software package. *J. Appl. Crystallogr.* **2013**, *46*, 544–549.

(7) Akahama, Y.; Kawamura, H. Pressure calibration of diamond anvil Raman gauge to 310 GPa. *J. Appl. Phys.* **2006**, *100*.

(8) Sharma, S.; Mao, H.; Bell, P. Raman measurements of hydrogen in the pressure range 0.2-630 kbar at room temperature. *Phys. Rev. Lett.* **1980**, *44*, 886.

(9) Kresse, G.; Furthmüller, J. Efficient Iterative Schemes for Ab initio Total-energy Calculations Using a Plane-wave Basis Set. *Phys. Rev. B* **1996**, *54*, 11169–11186.

(10) Kresse, G.; Joubert, D. From ultrasoft pseudopotentials to the projector augmented-wave method. *Phys. Rev. B* **1999**, *59*, 1758–1775.

(11) Perdew, J. P.; Burke, K.; Ernzerhof, M. Generalized Gradient Approximation Made Simple. *Phys. Rev. Lett.* **1996**, *77*, 3865–3868.

(12) Parrinello, M.; Rahman, A. Crystal Structure and Pair Potentials: A Molecular-Dynamics Study. *Phys. Rev. Lett.* **1980**, *45*, 1196–1199.

(13) Parrinello, M.; Rahman, A. Polymorphic transitions in single crystals: A new molecular dynamics method. *J. Appl. Phys.* **1981**, *52*, 7182–7190.

(14) Togo, A.; Chaput, L.; Tadano, T.; Tanaka, I. Implementation strategies in phonopy and phono3py. *J. Phys. Condens. Matter* **2023**, *35*, 353001.

(15) Togo, A. First-principles Phonon Calculations with Phonopy and Phono3py. *J. Phys. Soc. Japan* **2023**, *92*.

(16) Skelton, J. M.; Burton, L. A.; Jackson, A. J.; Oba, F.; Parker, S. C.; Walsh, A. Lattice dynamics of the tin sulphides SnS_2, SnS and Sn_2S_3: Vibrational spectra and thermal transport. *Phys. Chem. Chem. Phys.* **2017**, *19*, 12452–12465.
